# Refined Industrial Tannins via Sequential Fractionation: Exploiting Well-Defined Molecular Structures for Controlled Performance in Pickering Emulsions Costabilized with Chitin Nanofibrils

**DOI:** 10.1021/acssuschemeng.4c07769

**Published:** 2024-11-20

**Authors:** Weitong Wang, Ya Zhu, Monika Österberg, Bruno D. Mattos

**Affiliations:** † Department of Bioproducts and Biosystems, School of Chemical Engineering, Aalto University, P.O. Box 16300, Aalto FIN-00076 Espoo, Finland

**Keywords:** tannin, fractionation, solvent solubility, polyphenol materials, oil−water emulsion, interface stabilizing, biorefineries

## Abstract

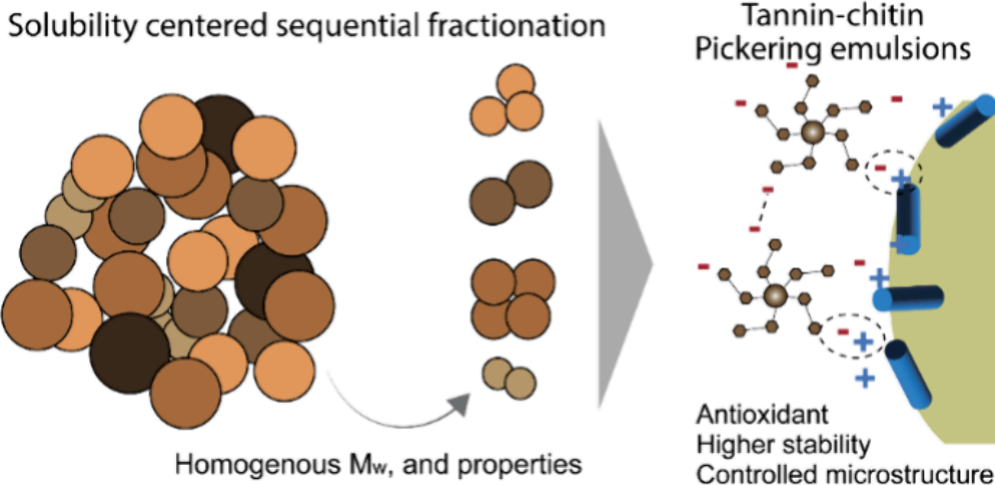

Tannins from *Acacia mearnsii* (black wattle) are one of the few industrially available sources of nonlignin polyphenols. The intrinsic chemical heterogeneity and high dispersity of industrial tannins complicate their use in applications where the reactivity or colloidal interactions need to be precisely controlled. Here, we employ a solubility-centered sequential fractionation to obtain homogeneous tannin fractions with a dispersity index lower than 2. The well-defined and homogeneous fractions were characterized using NMR and MALDI-TOF and were used to prepare Pickering emulsions by costabilization with chitin nanofibrils. We demonstrate that the emulsion droplet size and associated properties can be tuned by using tannin fractions of varied molar mass, which is a result of fine control over the tannin–chitin complexation interactions at the oil–water interface. In addition to enhancing emulsion stability, the addition of tannin to chitin-stabilized Pickering emulsions has proven to be a viable strategy for engineering the emulsion’s viscoelastic properties, as well as introducing antioxidative properties. Overall, we demonstrate a facile method to finely control the properties of industrial tannins and enable their customization to allow their utilization in high-performance multiphase systems.

## Abstract 2

Industrial tannins are fractionated, based on solubility parameter, into well-defined fractions that are used to control the properties of Pickering emulsions costabilized with chitin fibrils.

## 1 Introduction

Plant polyphenols are potential candidates for creating truly renewable and sustainable chemicals and materials, directly contributing to establishing a circular bioeconomy.^1,2^ Tannins, polyphenols extracted mostly from barks and shrubs, are water-soluble and low molar mass macromolecules that display strong metal-complexing capacity,^3,4^ reactivity toward aldehydes,^5^ as well as anti-inflammatory and antioxidant properties.^6^ Such variety of characteristics places tannins in a versatile position, in which it can be used in advanced materials or biomedical and food applications.^7^ Tannins can be divided into hydrolyzed (e.g., ellagitannins and gallotannins) and condensed molecules (e.g., proanthocyanidins), the latter being the most available and affordable group of molecules given their significantly higher yield in plants when compared to hydrolyzable molecules. Although millions of tons of condensed tannins are produced annually, their applications have been largely limited to animal feed and the leather industry.^8,9^ Using condensed tannins, as well as other natural polyphenols, in higher-value-added applications requires better defined molecules with known chemical structures. Tannins obtained at industrial scales have high DIs and chemical variability, thus displaying a wide range of responses as far as their antioxidant properties, adsorption behavior, and interfacial activity.^10−12^

Fractionation is a crucial and effective method for producing natural polyphenol fractions with improved homogeneity, controlled molar mass distribution, well-defined chemical compositions, and adjustable chemical functionality. Preparatory chromatography,^13−15^ selective precipitation,^16,17^ ultrafiltration,^18^ and sequential extraction-driven fractionation^19,20^ have been used to fractionate tannins. Among these, sequential fractionation shows the highest potential for industrial-scale implementation since it is based on simple extraction processes that can be easily scaled up. Sequential fractionation separates plant polyphenols by sequentially extracting the starting material with solvents of increasing hydrogen-bonding capability,^21,22^ which enables fractionation based on the solubility of the various components present in industrial tannin in an individual or mixed organic solvents. Soxhlet extraction, a common tool utilized in sequential fractionation, has been applied to efficiently fractionate hydroxyaromatic organic compounds, including lignin and tannin. Previous efforts on tannin purification approached the subject from a solvent polarity point of view; however, the obtained fractions were as heterogeneous as the starting precursors and there was not a clear correlation between solvent polarity and molar mass of the fraction.^19^ While these methods provide an effective platform for studying the physical, chemical, and functional properties of tannin fractions, their fraction outcomes are not well defined enough to justify their large implementation. Furthermore, it is ideal that a clear correlation between extraction solvent and tannin fraction exists, thus creating a fundamental understanding of the overall fractionation process and enabling control over the process outcome. However, most studies on tannin fractionation through sequential solvent fractionation have primarily concentrated on the chemical characterization of the isolated tannin fractions, often overlooking important aspects related to the interactions between solvent properties and characteristics of the constructed materials and systems. New fractionation parameters must be explored to systematically rationalize the process–property–performance relationship in potential applications for plant polyphenols.

Efficient utilization of plant polyphenols in advanced materials or systems, such as adhesives,^23^ foams,^3^ and hydrogels,^24^ relies on understanding and controlling their molecular structures. This is true for both lignin and tannins. Although typically lignin has been the flagship plant polyphenol for materials applications,^25−28^ tannins have higher solubility, which leads to easier pathways for modification (chemically or enzymatically), higher antioxidant activity than lignin, and greater potential for interfacial stabilization in multiphase systems.^29^ In this study, we propose a solubility-centered sequential fractionation method to produce several well-defined fractions from industrial tannins, which are used to trace a clear correlation between polyphenol and application properties. Our method uses a solvent series (acetonitrile, acetone, methanol, and ethanol) defined based on extensive tannin solubility pre-assessments. Each fraction is systematically analyzed, both quantitatively and qualitatively, to identify their chemical functionalities and macromolecular configurations, thus connecting the fractionation process to tannin fraction properties. Then, we rationalize the property–performance correlation between the tannin fraction properties with their performance in application by using Pickering oil-in-water emulsions as an application demonstrator. We use chitin nanofibrils (NCh) as the emulsifier,^30,31^ and the tannin fractions as a stability booster and functional component. We then quantify the effect of adding tannins on the emulsion properties, including physical stability, viscoelasticity, and antioxidant capacity.^32^ Understanding these properties and how they connect to the emulsion formulation is essential for selecting tannin fractions with tailored characteristics to meet the specific material requirements. Overall, the results of this study demonstrate a straightforward approach for obtaining tannin fractions with well-defined characteristics, which can be efficiently utilized in the development of new and improved biobased systems. Additionally, we traced a clear correlation between the physicochemical characteristics of the fractions and their application performance, which is crucial for advancing the development of high-performance tannin-based systems.

## 2 Experimental Section

### 2.1 Materials

Industrial tannin (IT) was extracted from the bark of black wattle (*Acacia mearnsii*) and provided by TANAC S/A Brazil. Chitin nanofibrils (NCh), with a zeta potential of +60 mV and a protonation degree at pH 3 of 0.99 (following the Henderson–Hasselbalch equation), were sourced from fresh crabs (*Callinectes sapidus*) that were acquired from the local market in Helsinki Harbor, Finland. The methodologies, encompassing purification, and deacetylation adhered to previously documented protocols,^33,34^ with detailed information available in the Supporting Information.

Acetonitrile (ACN), acetone (ACE), methanol (MeOH), ethanol (EtOH), dimethyl sulfoxide-d6, acetic acid, isooctane, 2-propanol, 1-butanol, 2,5-dihydroxybenzoic acid (2,5-DHB), and Folin-Ciocalteu reagent were of analytical grade and purchased from Merck (Sigma-Aldrich, Finland). Other reagents including Na_2_CO_3_, HCl, NaNO_2_, AlCl_3_, NaOH, FeSO_4_, BaCl_2_, H_2_O_2_, gallic acid hydrate, catechin hydrate, vanillin, ammonium thiocyanate, trichloroacetic acid, thiobarbituric acid, and 1,1,3,3-tetramethoxypropane were purchased from TCI (Finland). All chemicals were free of further purification. Rapeseed oil was acquired from a local market in Helsinki, Finland. Deionized water (DIW), generated using a Millipore Synergy UV unit (18.2 MΩ·cm), was utilized throughout the experiments.

### 2.2 Fractionation Process

**Figure 1:**
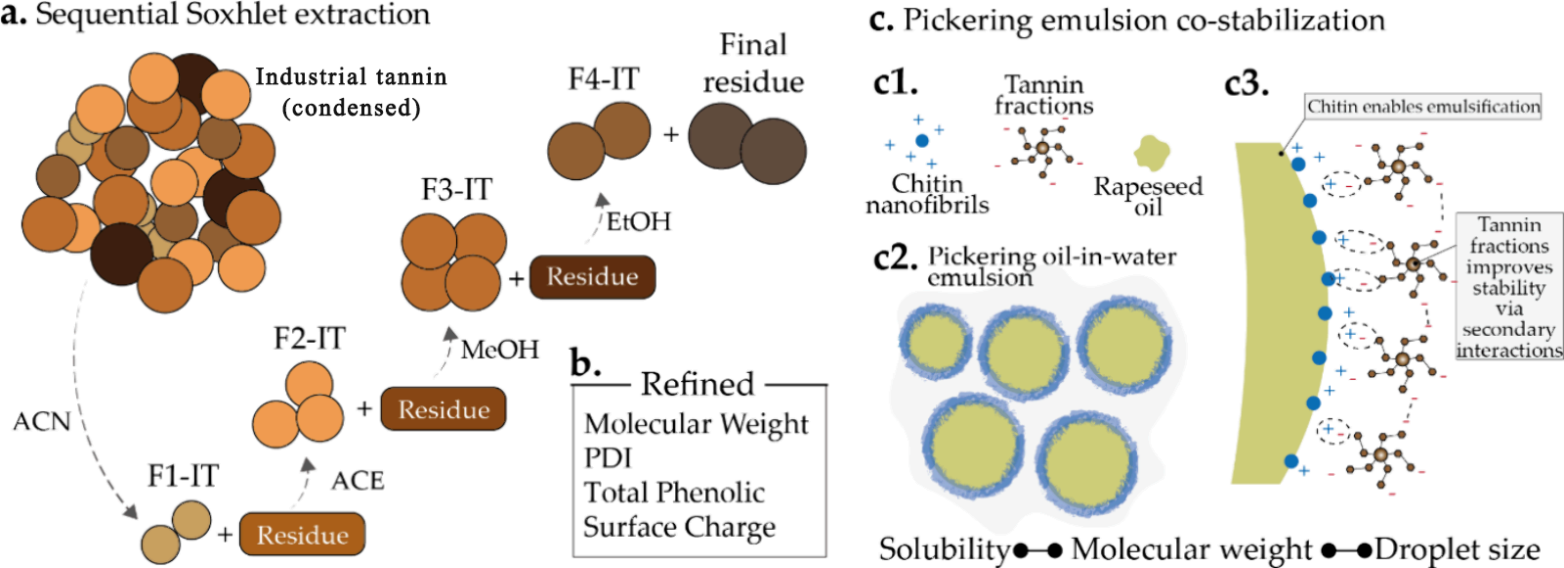
Refining of industrial condensed tannins by sequential extraction and use of well-defined fractions in Pickering emulsion costabilization with chitin nanofibrils. (a) Solubility-driven sequential Soxhlet extraction yields several fractions with (b) narrower molecular weight distribution, tuned phenolic content, and surface charge. (c) Pickering emulsions are prepared using chitin nanofibrils as the emulsification agent, refined condensed tannins as the stabilizer, and rapeseed oil as the noncontinuous phase (c1). (c2) Stable Pickering emulsions are formed with controlled droplet size. (c3) Stability is driven by electrostatic interactions between chitin and tannins, as well as by multiple hydrogen bonding among all biopolymers.

Initially, 25 g of IT was placed in a cellulose extraction thimble (Whatman, Merck Finland). Fractionation was carried out in a 250 mL Soxhlet extraction tube using ACN, ACE, MeOH, and EtOH as solvents (350 mL for each solvent; Figure 1a). Each residue obtained from the preceding step was utilized as the starting material for the subsequent step. Each step was performed for eight cycles (a cycle is considered when the solvent siphons in the Soxhlet extraction tube). The resulting fractions (Fx-ITs) were labeled as F1-IT, F2-IT, F3-IT, and F4-IT. The yield of each fraction was calculated using eq 1:
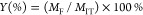
1

*M*_F_ = final mass of each fraction after fractionation (g); *M*_IT_ = initial mass of IT (g).

### 2.3 Emulsion Preparation

Chitin nanofibrils (NCh) were dispersed in DIW and adjusted to a specific concentration. The NCh suspension and tannin solutions (IT, F1-IT, F2-IT, F3-IT, and F4-IT) were mixed at 25 °C with a pH of 3 (adjusted by acetic acid) to prepare oil/water emulsions. Each tannin solution was added to the chitin suspension at different concentrations (50, 25, 12.5, 6.25, and 3.125% over the mass of chitin), mixed, and vortexed for 30 s. Subsequently, mixtures of NCh (8.5 mL) and tannin (0.5 mL) were combined with rapeseed oil (1 mL) and vortexed for an additional 30 s. The oil–water mixture underwent emulsification in an ice bath via tip sonication using a titanium tip sonicator (Sonifier 450, Branson Ultrasonics Co., Danbury, Connecticut), with the power level set at 10% using alternating on–off cycles (120 s total, 3 s on, 2 s off, respectively).

### 2.4 Characterization of Tannin Fractions

All quantitative analyses were carried out in triplicate.

#### 2.4.1 Total Phenolic Content (TPC)

The samples were initially dissolved in a 50% MeOH solution. Subsequently, 5 μL of sample solution was mixed with 195 μL of DIW, followed by the addition of 25 μL of Folin–Ciocalteu reagent. After reacting for 6 min, 75 μL of 7 wt % Na_2_CO_3_ solution was added, and the mixture was stored in the dark for 2 h. Finally, the absorbance was measured at 710 nm, and the TPC values were expressed as milligrams of gallic acid equivalents (mg of GA/g).

#### 2.4.2 Condensed Tannin Content (CTC)

For the measurement of CTC, 10 μL of sample solution was combined with 200 μL of MeOH (containing 4 wt % vanillin). Following this, 100 μL of concentrated HCl was added, and the mixture was allowed to react for 15 min. Subsequently, the absorbance was measured at 550 nm, and CTC values were expressed as milligrams of catechin equivalents (mg of CE/g).

#### 2.4.3 Total Flavonoid Content (TFC)

For the measurement of TFC, 30 μL of sample solution was mixed with 180 μL of DIW, followed by the addition of 10 μL of 5 wt % NaNO_2_ solution. After reacting for 6 min, 20 μL of 10 wt % AlCl_3_ solution was added, and the reaction continued for another 6 min. Subsequently, 60 μL of 4 wt % NaOH solution was added, and the mixture was allowed to stand for 15 min. Afterward, the absorbance was measured at 525 nm, and TFC values were expressed as milligrams of catechin equivalents (mg CE/g).

#### 2.4.4 Gel Permeation Chromatography

The molecular weight and distribution of both IT and tannin fractions were evaluated using gel permeation chromatography (GPC) with an Agilent 1100 HPLC system equipped with a PSS MCX 300 × 8 mm column (Agilent, USA). Elution was performed with a 0.1 M NaOH solution. Prior to analysis, both industrial tannins and tannin fractions were dissolved in a 0.1 M NaOH solution and filtered through a 0.45 μm filter membrane.

#### 2.4.5 MALDI-TOF

Structural analysis was conducted by utilizing a Bruker Maldi UltrafleXtreme TOF instrument (Bruker, USA). For this analysis, 50 mg/mL sample solutions were blended with the 20 mg/mL matrix solution (2,5-DHB was utilized as the matrix) at a volumetric ratio of 1:3 in a 30% ACE solution, and then a 1 μL mixture was spotted on the steel target for testing after being air-dried.

#### 2.4.6 NMR Analysis

Spectra of ITs and each fraction were acquired using a Bruker NMR Spectrometer AV III 400 spectrometer (400 MHz, Bruker, USA). Prior to analysis, samples were dissolved in dimethyl sulfoxide-d6. We used the following parameters for the HSQC experiments: acquisition from 15 to 0 ppm in F2 (^1^H) by using 2048 data points for an acquisition time of 128 ms, and 210–0 ppm in F1 (^13^C) by using 512 increments (F1 acquisition time 11.6 ms) of 48 scans with a 1.5 s interscan delay. HSQC cross-peaks were assigned by comparing the spectra to the authentic monomeric and dimeric samples. Complementarily, we have used quantitative ^31^P NMR, whose detailed methodologies for sample preparation and experiments are found in the Supporting Information.

#### 2.4.7 FTIR Analysis

Functional groups of IT, Fx-ITs, and the residue were analyzed under the attenuated total reflectance (ATR) mode using Fourier transform infrared spectroscopy (FTIR, PerkinElmer, USA) over the range 450–4000 cm^–1^.

#### 2.4.8 Element Analysis

The content of inorganic elements in ITs and its fractions was semiquantitatively analyzed using SEM-EDS (Zeiss Sigma VP, Germany). Carbon, hydrogen, nitrogen, oxygen, and sulfur were analyzed in a Thermo Flash Smart CHNSO Elemental Analyzer (USA).

### 2.5 Characterization of Pickering Emulsions and NCh–Tannin Adsorption

#### 2.5.1 Zeta Potential

Each tannin fraction, tannin–NCh complex, and emulsion had their zeta potential acquired using a Zeta-sizer analyzer (Nano ZS90, Malvern, UK).

#### 2.5.2 Atomic Force Microscopy

The aspect ratio and overall morphology of NCh were assessed using atomic force microscopy (MultiMode 8, Bruker, USA) equipped with a NCHV-A probe (40 N/m, 320 kHz) in tapping mode and no image modification after analysis.

#### 2.5.3 Quartz Crystal Microbalance with Dissipation (QCM-D)

The adsorption of tannin fractions on chitin nanofibrils was monitored using a QSense E4 (Biolin Science, Sweden) using silicon sensors (QSX 303, Biolin Science, Sweden). Silicon sensors were initially immersed in 10 wt % NaOH solution for 1–2 s (to prevent corrosion of the silicone coating), rinsed thoroughly with DIW under ultrasonic bath for 20 min to eliminate residual NaOH, dried with nitrogen gas, and then subjected to UV/ozone conditions (UV/Ozone ProCleaner, BioForce Nanosciences, Germany) for 15 min. After the sensor pretreatment, 150 μL of a 0.1 wt % chitin solution was deposited onto the sensor surface using a spin coater (WS-650SX-6NPP/LITE, Laurell Technologies, USA) to create a uniform thin film. During QCM-D measurements, the concentration of injected tannin and tannin fraction solutions were 0.1 wt %, and the injection flow rate was set at 100 μL/min. The adsorption mass of tannin on NCh film was proportional to the decrease in the resonance frequency, which can be calculated using eq 2 (Sauerbrey equation):
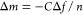
2where Δ*f* is the frequency of the crystal in Hz, Δ*m* is the adsorption mass, *C* is the sensitivity factor constant (−0.177 mg/m^2^·Hz), and *n* is the number of overtones (*n* = 3, 5, 7, 9, 11, 13), and the third overtone was used here.

#### 2.5.4 Emulsion Droplet Size

The oil droplet size distribution of each emulsion was measured by using a laser diffraction light scattering instrument (Mastersizer 3000, Malvern Instruments, UK). Emulsion (4 mL) was added to DIW (800 mL); the refractive index of the oil phase was 1.468, and the refractive index of the water phase was 1.33. The surface weighted mean diameter (*d*_(3,2)_) was calculated based on eq 3:
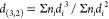
3where *n_i_* is the number of droplets and *d_i_* is the droplet diameter.

#### 2.5.5 Rheology Studies

Rheological measurement was conducted with a rheometer (MCR 302, Anton Paar, Germany) equipped with a parallel plate (PP25) and a gap fixed at 1 mm; samples were presheared at a shear rate of 10 s^–1^.

#### 2.5.6 Emulsion Physical Stability

The physical stability of emulsions was monitored using a Turbiscan MA2000 (Formulaction, France). The apparatus comprises a detection head equipped with a near-infrared light source (880 nm) that scans the length of the sample, acquiring transmission and backscattering data every 40 μm. The fresh emulsion was stirred evenly and transferred to test tubes immediately. Then, the emulsion was scanned with the instrument every 30 min over 24 h.

#### 2.5.7 Three-Phase Contact Angle of the Tannin–NCh Complex

The wetting properties of the tannin–NCh complex were tested by the Optical Tensiometer (Theta Flex, Biolin, Sweden). The tannin–NCh complex and NCh were immersed into a quartz container containing rapeseed oil, and then 2 μL of DIW was dropped onto the surface of the flake to record the contact angle.

#### 2.5.8 Microstructure of Emulsions

The micromorphology of oil droplets in emulsions was observed by an Olympus microscope (Olympus, BX53M, Japan).

#### 2.5.9 Antioxidant of Emulsions

The Pickering emulsions were stored at 45 °C and analyzed for both primary oxidative products (lipid hydroperoxides) and secondary oxidative products (2-thiobarbituric acid reactive substances, TBARS) to evaluate lipid oxidation, in accordance with a previous study.^35^ For the measurement of lipid hydroperoxides, 0.2 mL of emulsion was mixed with 1.5 mL of extracting solvent (1.125 mL of isooctane and 0.375 mL of 2-propanol) and vortexed for 2 min. Subsequently, a 200 μL sample was taken and added to 2.8 mL of MeOH:1-butanol (2:1, v/v) mixture. 50 μL of 3.94 M ammonium thiocyanate solution and 50 μL ferrous iron solution (prepared by mixing 0.132 M BaCl_2_ and 0.144 M FeSO_4_ solution in equal amounts) were further added to the sample. After 25 min reaction, the absorbance of samples was measured at 510 nm using a UV spectrophotometer (Shimadzu UV-2550, Japan), and concentrations of lipid hydroperoxides were quantified using the standard curve generated from H_2_O_2_.

For the measurement of TBARS, 1 mL of emulsion was mixed with 2 mL of TBA reagent solution (containing 15 wt % trichloroacetic acid and 0.375 wt % thiobarbituric acid in 0.25 M HCl) and boiled for 15 min. Samples were taken for measurement after being cooled to room temperature. The absorbance was measured at 540 nm, and the TBARS content was evaluated according to a standard curve produced from 1,1,3,3-tetramethoxypropane. All measurements, encompassing the evaluation of primary and secondary reaction products, were conducted in triplicate throughout the 21-day storage period, with assessments performed every 3 days.

## 3 Results and Discussion

### 3.1 Characterization of Industrial Tannin and Its Fractions

**Figure 2:**
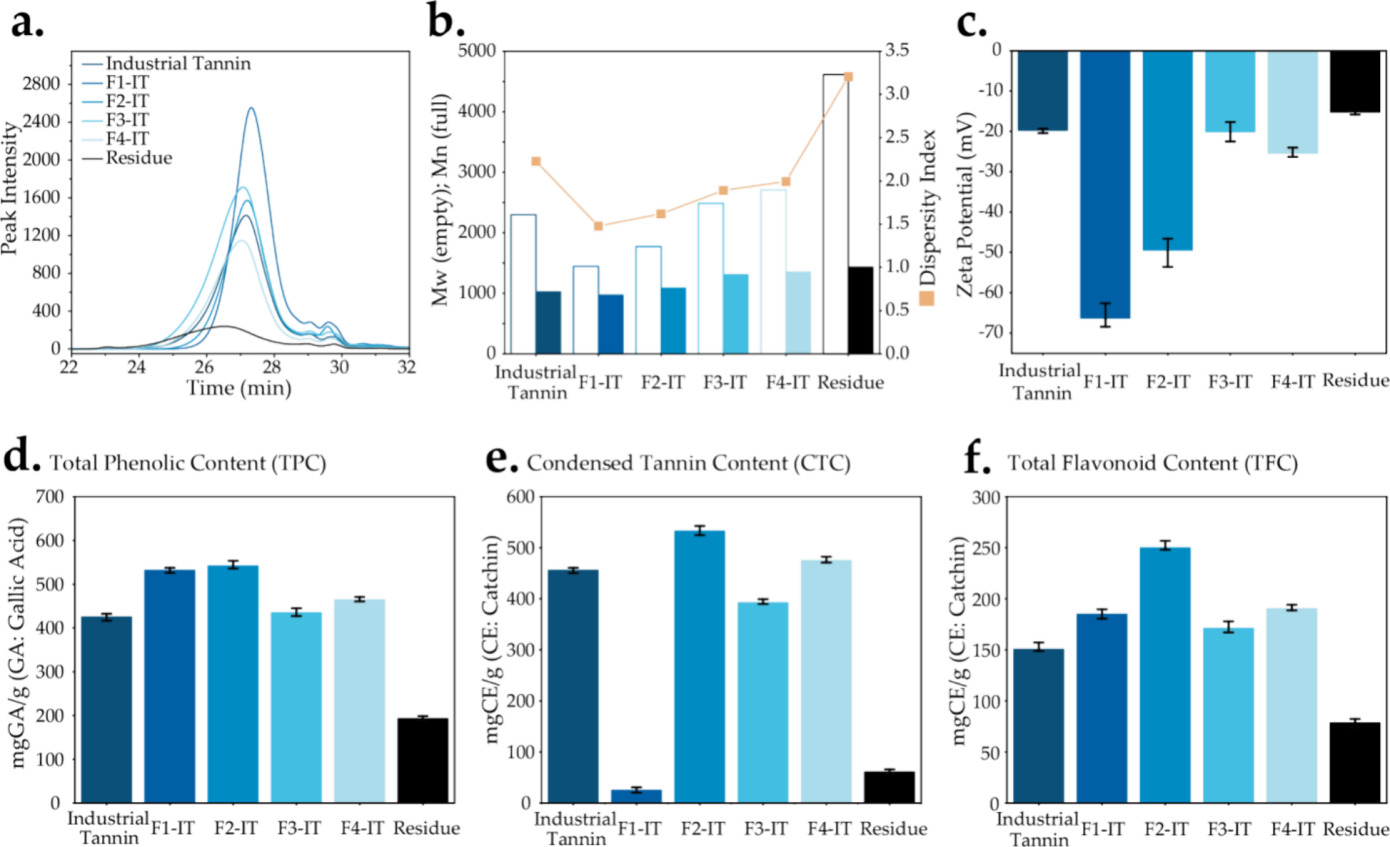
Chemical characterization of IT and tannin fractions. (a) Gel permeation chromatography (GPC) curves. (b) Weight-averaged molecular weight (*M*_w_), number-averaged molecular weight (*M*_n_), and dispersity index. (c) ζ-Potential of IT, F1-IT, F2-IT, F3-IT, F4-IT, and residue at pH 6.73. (d) Total phenolic content (TPC), (e) condensed tannin content (CTC), and (f) total flavonoid content (TFC) of each fraction, starting precursor, and residue material.

Prior to employing the Soxhlet method for fractionating ITs into distinct fractions, we conducted a preliminary assessment of tannin solubility in various solvents (Figure S1). Subsequently, we evaluated the weight-average molecular weight (*M*_w_) and number-average molecular weight (*M*_n_) (Figure 2a), and DI for both soluble and insoluble portions (Table S1). The selection and sequence of our solvents were motivated by their distinct solubility capacity toward ITs. By employing a set of four selected solvents (ACN, ACE, MeOH, and EtOH), *M*_w_ of soluble parts exhibited a sequential increase corresponding to the enhanced solubility of tannins in each given solvent (Figure S1b). Based on an increased dissolution capacity, the following fractionation sequence was adopted: ACN → ACE → MeOH → EtOH. Extraction yields and *M*_w_ are presented in Table 1, and there is not a clear correlation between them. MeOH and EtOH are both extremely good solvents for tannins, but EtOH is slightly better. Thus, placing EtOH at the end of the fractionation allows us to have another well-defined fraction after the extraction with MeOH. This clearly indicates that solubility is a better parameter than the solvent polarity as far as tannin fractionation. Given its efficiency for selectively dissolved tannins, EtOH is also a viable option to simply purify tannin from residues like ashes, sugars, and insoluble polyphenols. Interestingly, fractions extracted with later solvents showed limited solubility in the earlier solvents. For instance, F3-IT could not dissolve in ACE or ACN but was totally soluble in EtOH. This phenomenon can be explained by considering the preference of different compounds in ITs to dissolve in specific solvents. Some compounds exhibit solubility in multiple solvents, while others dissolve exclusively in only a few solvents. Consequently, our fractionation process enabled a low DI, which may be challenging to achieve in a binary-solvent system via solely adjusting the concentration (e.g., THF–water for lignin fractionation). By employing solvents with varying solubilities for sequential fractionation, specific components can be efficiently separated, akin to a “sieving” process. This approach avoids interference between solvents in sequential fractionation steps, enhancing the overall efficiency of the process.

**Table 1:** Fraction Yields and Molecular Weights Extracted Using Different Solvents

	F1-IT	F2-IT	F3-IT	F4-IT
extraction solvent	ACN	ACE	MeOH	EtOH
yield (%)	5.89	25.39	54.74	1.64
*M*_w_^a^	1445.5	1772.7	2490	2710.5

a Weight-average molecular weight.

The DIs of tannin fractions ranged from 1.47 to 1.99, with a slight increase at each step of the fractionation (Figure 2b). The DIs obtained in this process are much better than previous values, adopting, for example, polarity-driven solvent selection, which led to fractions with DIs ca. 3–4 (Table S2). The absolute value of the ζ potential decreases with increasing *M*_w_ (Figure 2c). The high variability in ζ-potential of the different fractions factors in potential applications in colloidal systems or that aims at exploiting the reactivity of the obtained tannin fractions. This suggests that F1-IT, with a ζ-potential equal to −60 mV, may be more colloidally stable and exhibit superior reactivity toward cationic entities.^36^

The chemical composition of each fraction and ITs was assessed by quantifying the TPC, CTC, and TFC (Figure 2d–f). Our results show that both phenolic and flavonoid compounds are soluble in ACN, ACE, MeOH, and EtOH. Remarkably, F2-IT (after ACN and ACE) exhibited the highest TPC, CTC, and TFC contents, measuring 542.6 mg GA/g, 533.4 mg CE/g, and 250.2 mg CE/g, respectively. The low CTC observed in F1-IT (25.8 mg of CE/g) indicates that condensed tannins are poorly soluble in ACN but highly soluble in ACE and alcohol. The varying chemical compositions also impart distinct colors to the different tannin fractions (Figure S2), which can be an asset in several applications, e.g., within cosmetics and food. The lower content of TPC (193.8 mg of GA/g), CTC (61.6 mg of CE/g), and TFC (79 mg of CE/g) in the residual material shows that phenolic and flavonoid compounds can be effectively separated and retained in different fractions. FTIR characteristic peaks (Figure S3) were observed around 3265 cm^–1^ (O–H stretching), 1443–1601 cm^–1^ (aromatic C_6_), 1304 cm^–1^ (phenol −OH), and 845 cm^–1^ (aromatic C–H), indicating an abundant polyphenol content in the tannin fractions.^37^ In particular, F2-IT displayed intense peaks at 1694 cm^–1^ due to the carboxyl groups (C=O) and 1255 cm^–1^ due to C–O stretching of the carboxyl group. Moreover, F1-IT showed pronounced peaks at 1601 cm^–1^ (C=C aromatic skeletal vibration), 1507 cm^–1^ (aromatic C_6_), and 1304 cm^–1^ (phenol −OH).^38^ The ^31^P NMR data (Table 2) confirms the content of polyphenols across the distinct fractions, by highlighting their distinguished hydroxyl functional groups (Figure S4).^39^ The absorption in the ring C region (146.0–145.0 ppm) indicates a significant presence of aliphatic OH, mainly attributed to carbohydrates. The oxidative pattern of ring B is characterized as catechol (140.2–138.8 ppm), with the presence of some pyrogallol units (144.0–140.2 ppm). Additionally, the ring B region can be divided into two subregions: 142.5–141.8 and 141.5–141.0 ppm for *o*-disubstituted phenolics, and 139.4–137.9 ppm for *o*-monosubstituted hydroxyphenyl groups. The characteristics of ring A are identified through the integration of *o*-unsubstituted (137.9–137.4 ppm) and *o*-substituted phenolic groups (138.8–137.9 ppm). This analysis indicates that F1-IT and F2-IT are primarily composed of hydrolyzable tannins, accounting for 31.28% of the total product (Table 2). Much less difference is observed between F3-IT and F4-IT, suggesting that although the solubility of IT in MeOH and EtOH is different, they are both similar alcohol extracts. For the insoluble residue (after the four extractions), only two distinct peaks were observed at 1601 and 1023 cm^–1^, which can be assigned to the C=C aromatic skeletal vibration and C–O–C asymmetric vibrations. These spectral patterns suggest that the insoluble residues are mostly carbohydrates, with only a very limited content of polyphenols. Meanwhile, elemental analysis (Table S3) shows an increase in the carbon content of fractions compared to tannin and residue, indicating that the polyphenols are continuously dissolved and effectively retained in fractions during the fractionation process, demonstrating a concentration of such carbon-rich molecules.

**Table 2:** Phenolic Groups (mmol/g) in Tannin and Fx-ITs as Evaluated by ^31^P NMR

		IT	F1-IT	F2-IT	F3-IT	F4-IT
ring A	*o*-unsubstituted phenolic OH	1.04	1.49	1.23	0.65	0.79
	*o*-substituted phenolic OH	1.94	2.68	2.38	1.37	1.62
ring B	catechol	2.36	2.89	2.49	1.71	1.99
	pyrogallol	2.56	2.53	2.7	2.02	2.2
ring C	aliphatic OH	0.89	1.1	0.89	0.61	0.72

**Figure 3:**
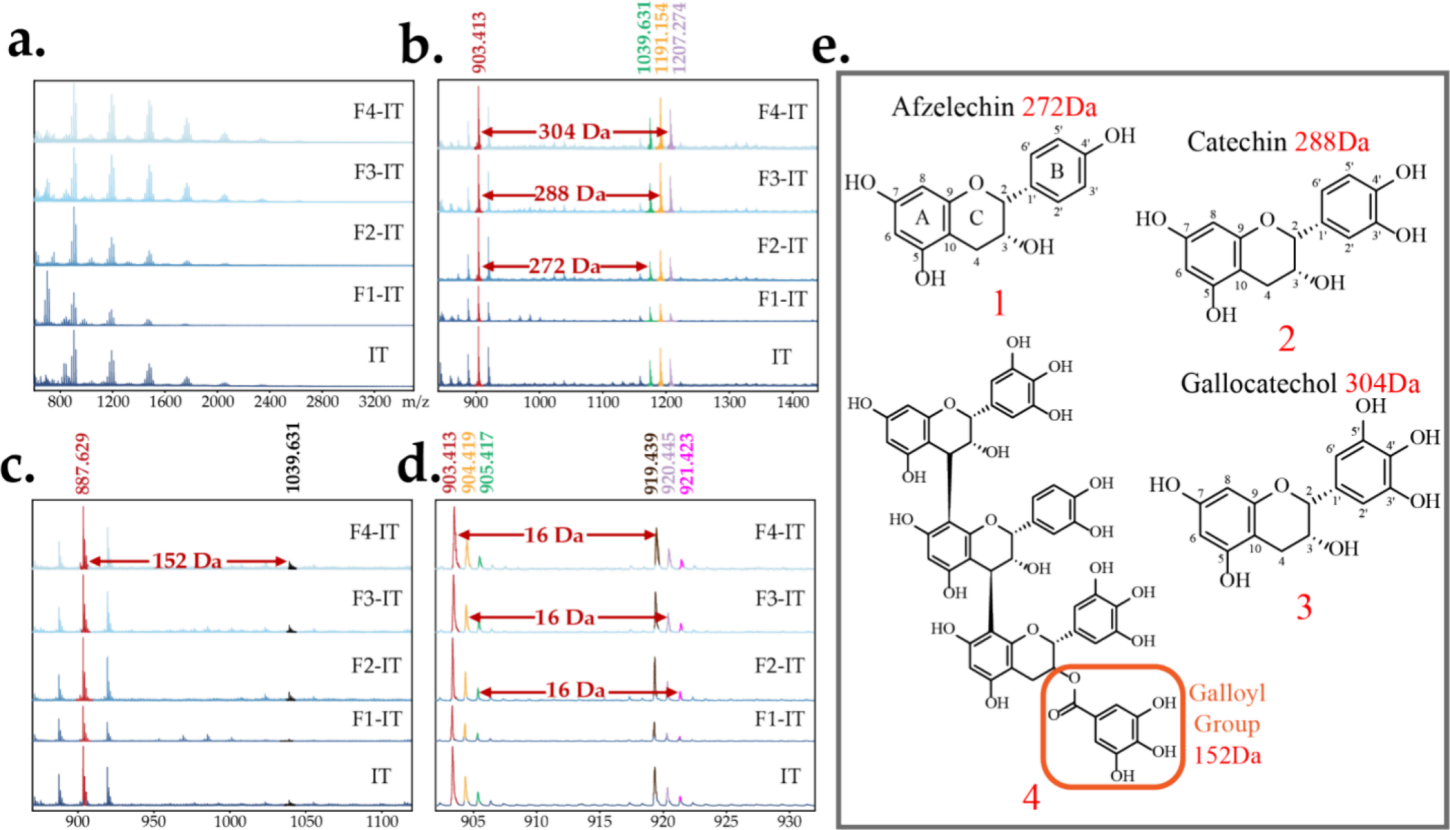
MALDI-TOF spectra were employed to further characterize (a) IT, F1-IT, F2-IT, F3-IT, and F4-IT, with (b) detailing the 840–1440 Da range and indicating relevant 304, 288, and 272 Da repeating units, (c) detailing the 840–1140 Da range and indicating the 152 Da repeating units, and (d) detailing the 902–932 Da range and indicating the 16 Da repeating unit. Additionally, the structural representations of (e1) afzelechin, (e2) catechin, and (e3) gallocatechol along with depictions of (e4) show the binding configuration of the galloyl group.

Next, we employed MALDI-TOF and ^1^H–^13^C HSQC NMR to characterize the macromolecular configurations of the tannin fractions. MALDI-TOF was used to determine the polymer chain length and the sequential arrangement of monomer units within individual chains,^40^ as illustrated in Figure 3a–d. Tannin fractions and ITs (raw material) exhibit similar heteropolymer structures with spectra showing evenly spaced peak signals. The peaks at higher mass signals get stronger with increasing *M*_w_ from F1-IT to F4-IT, thus indicating a higher *M*_w_ that is a result of more repeating monomeric structures and elevated polymerization degree (PD) for these compounds. In Figure 3b, repeating units of 304, 288, and 272 Da correspond to gallocatechin, catechin, and afzelechin monomers, respectively. The strongest signals, spaced by 288 Da, indicate that catechin is the primary constituent in IT and each of its fractions.^15^ The repeating unit of 152 Da corresponds to galloyl (Figure 3c), which is indicative of mass signals associated with condensed tannins. This observation is linked to the addition of one galloyl group at the heterocyclic C ring (C-3 position).^41^ Additionally, paired peaks separated by 16 Da were detected (Figure 3d), indicating the loss or addition of a hydroxyl group at the B-3′ or B-5′ position.

**Figure 4:**
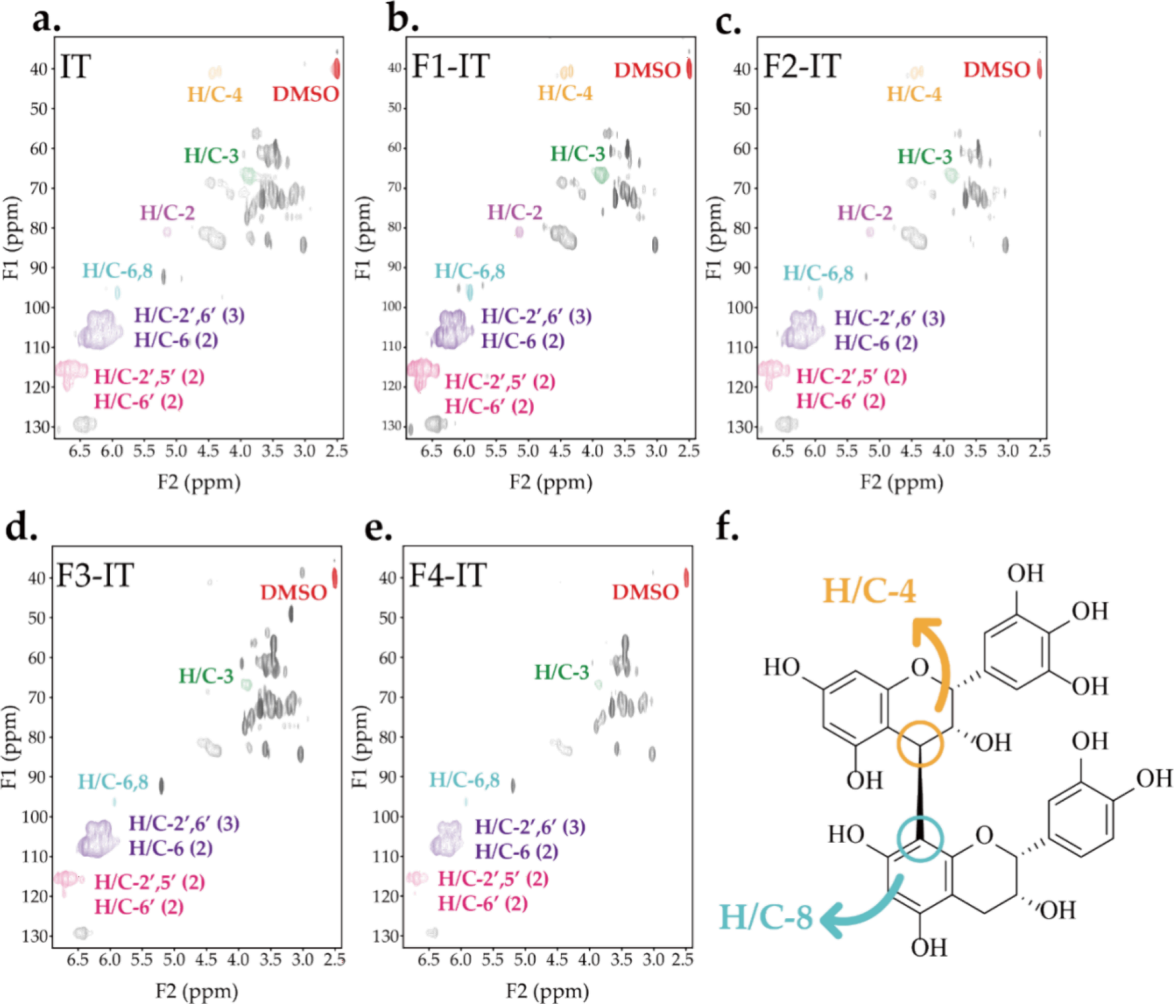
Chemical structures of industrial tannins and its fractions were elucidated through ^1^H–^13^C HSQC NMR spectra in (a) IT, (b) F1-IT, (c) F2-IT, (d) F3-IT, and (e) F4-IT, with the relevant cross-peak marked. (f) Structural representations of the binding form of the multimer.

HSQC NMR spectra are presented in Figure 4a–e. Excluding the cross-peak of DMSO-d6 at (2.5, 40.0), specific characteristic cross-peaks corresponding to notable H/C interactions are identified at distinct chemical shifts in ppm. These include H/C-3 (3.86, 66.78), H/C-4 (4.43, 40.98), H/C-2 (5.12, 80.97), H/C-6 (2, catechin), H/C-2′,6′ (3, gallocatechol) (6.21, 106.13), and H/C-6,8 (5.91, 96.45). Additionally, H/C-2′,5′ (2, catechin) and H/C- 6′ (2, catechin) (6.71, 115.41) are marked. The notation H/C-6 (2, catechin) refers to the hydrogen atom at position A-6 of the catechin structure, while H/C-2′,6′ (3, gallocatechol) refers to the hydrogen atoms at positions B-2′ and B-6′ of the gallocatechol structure. The HSQC NMR spectra of the ITs and its fractions (Fx-Its) revealed signals in the cross-peaks H/C-2′,6′ (3, gallocatechol), H/C-6 (2, catechin), and H/C-2′,5′ (2, catechin), indicating that catechin and gallocatechol are predominant in all samples. Furthermore, strong signals of H/C-8 and H/C-4 cross-peaks are evident in fractions F1-IT and F2-IT. This suggests that F1-IT and F2-IT exhibit a lower PD. Weak or unobserved signals of the resonances of H/C-4 and H/C-8 cross-peaks usually indicate high PD since the polymerization takes place between the positions C-4 and C8, as shown in Figure 4f.^42^ Overall, the combined analysis of the HSQC NMR and MALDI-TOF confirmed the presence of three primary structural units (afzelechin, catechin, and gallocatechol) across all tannin structures and also present a perspective for determine the PD of tannins via structure changes.

### 3.2 Application of Tannin Fractions in Pickering Emulsions Costabilized with Chitin Nanofibrils

Chitin nanofibrils (NCh) are effective to stabilize Pickering oil-in-water emulsions, given their highly positive surface charges and partial hydrophobicity.^43^ However, NCh alone cannot meet the antioxidant demands for some food or cosmetic applications, and a low NCh content in emulsions could lead to stratification over time. Therefore, we used NCh as the emulsifier in oil-in-water Pickering emulsions and incorporated tannin fractions to (i) control the emulsion microstructure and enhance its stability and (ii) to introduce antioxidant properties.

**Figure 5:**
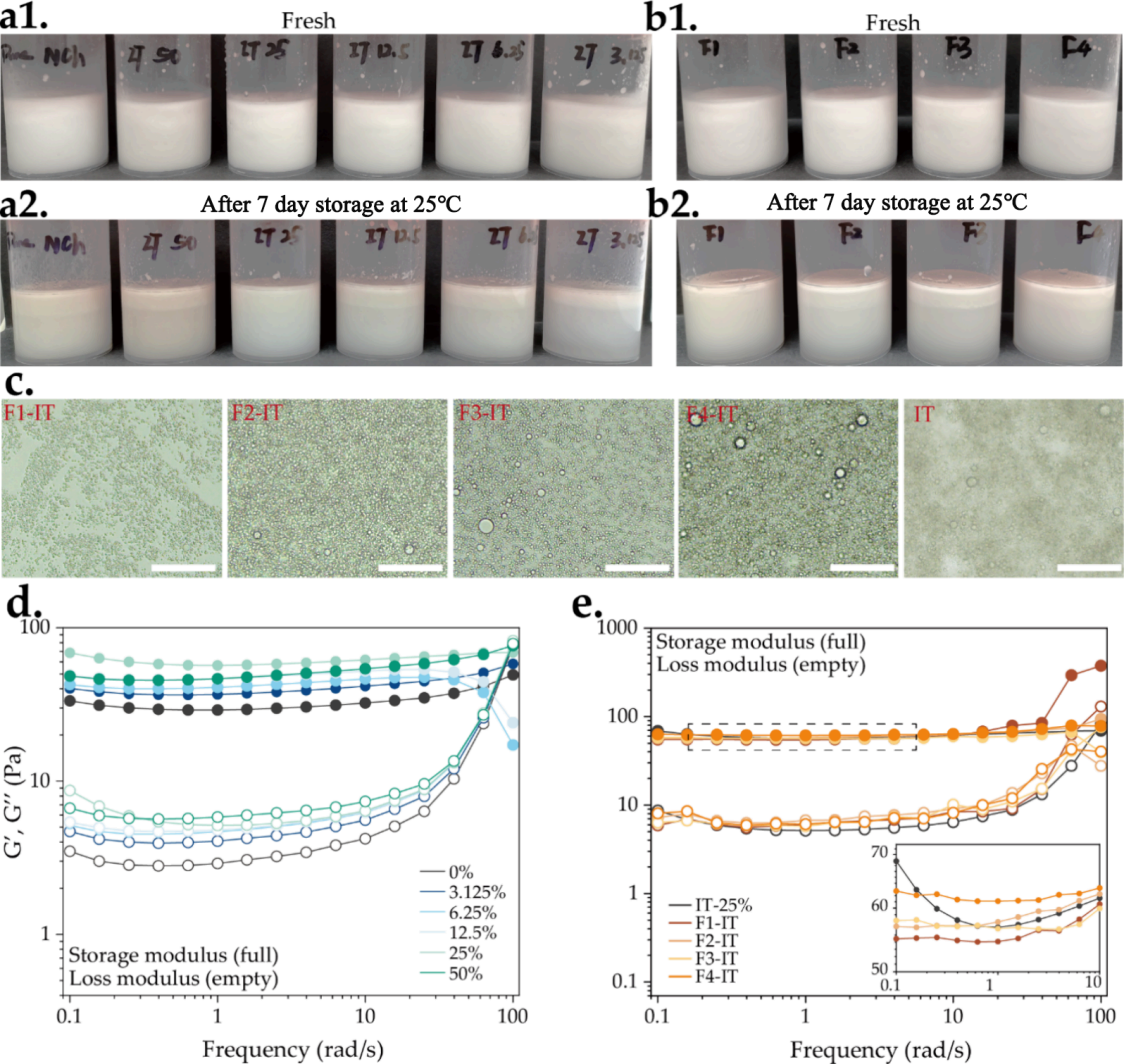
Appearance of fresh Pickering emulsions prepared with (a1) varying contents of IT (0, 50, 25, 12.5, 6.25, and 3.125 wt %, from left to right) and with (b1) Fx-ITs at 25 wt % (F1-IT, F2-IT, F3-IT, and F4-IT, from left to right) and emulsions of (a2) varying contents of IT and (b2) different Fx-IT storage after 7 days. (c) Micromorphology (scale bar 100 μm). Storage and loss modulus of (d) emulsion with various mass ratios of IT to chitin (0–50 wt %) and (e) emulsion stabilized by different fractions.

Pickering emulsions with long-term stability were produced by controlling the tannin–NCh ratio as well as tannin fractions, while keeping the oil fraction fixed at 10%. We observed no discernible phase separation in the tannin–NCh emulsion prepared with high NCh content (0.7 wt %) for a period of 45 days of storage at ambient temperature (Figure S6). Then, we reduced the NCh concentration to 0.07 wt % in order to more clearly observe the effects of adding ITs and their fractions on the emulsion properties along the 7 days. We found that tannin positively affected the emulsion stability. As the amount of tannin increased, the emulsion exhibited progressively increased stability during 7 days of storage (Figure 5a). However, when the IT concentration reached 50 wt %, it negatively affected stability, resulting in a clear separation between the top and bottom parts. Additionally, the thickness of the emulsified layer at the top of some emulsions remained significantly stable, especially when costabilized by F1-IT and F2-IT (Figure 5b).

To provide a data-driven demonstration of tannin’s role in stabilizing emulsions, we first studied the rheological properties of the emulsions (Figure 5d,e). The addition of increased amounts tannin, from 3.125 to 50 wt % over the NCh mass, modified the viscoelasticity of the Pickering emulsions. The most visible changes were observed after varying the tannin content, and minor changes took place after varying the fraction type. All emulsions had *G*′ (storage moduli) > *G*′′ (loss moduli) across the 0.1–10 rad s^–1^, with *G*″ increasing rapidly until the emulsion yielded at ca. 50 rad s^–1^. This predominantly elastic behavior is often observed in emulsions with interfaces stabilized by polyphenol and nanoparticle complexes, demonstrating a weak gel-like viscoelastic network.^44−46^ Both *G*′ and *G*″ of tannin–NCh-stabilized emulsions were higher than those of pure NCh, suggesting that the addition of tannin forms a more closely packed and stronger tannin–NCh complexed network structure. Elevating the tannin content in emulsions led to a continuous increase in *G*′, reaching its peak when the IT mass comprised 25 wt % of NCh. Then, *G*′ declined as the IT mass increased to 50 wt %. This indicates that the oil–water interface saturates already at 25 wt % of tannin, with a subsequent adsorption of tannin weakening the interface by developing competitive interactions. Positively charged particles can effectively stabilize rapeseed oil, which usually has negatively charged fatty acids. Therefore, with a high content of polyphenols (50 wt %) adsorbed on the positively charged NCh can reverse/decrease its charge, cause a poor stability of the emulsion, and finally reduce its elastic components.

**Figure 6:**
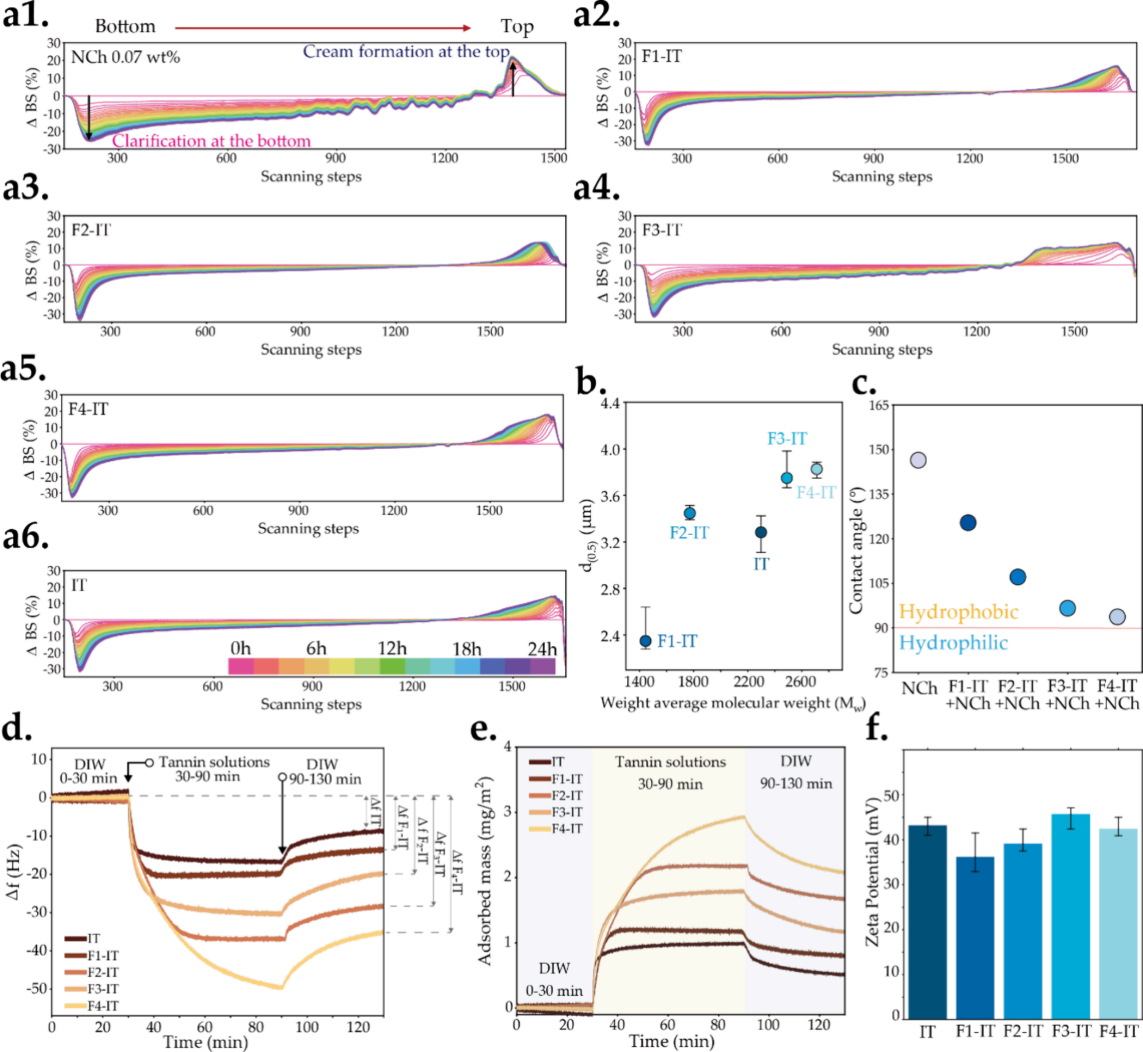
Turbiscan delta backscattering (ΔBS) graph of emulsions stabilized by (a1) pure NCh, and NCh costabilized with (a2) F1-IT, (a3) F2-IT, (a4) F3-IT, (a5) F4-IT, and (a6) IT at 25 wt % of NCh. (b) Oil droplet size of emulsions stabilized by different fractions. (c) Water contact angle on different tannin–NCh complex surfaces under oil. (d) QCM-D profiles displaying the changes in Δ*f* caused by adsorption of different fractions on the NCh surface. (e) Adsorption capacity of IT and Fx-ITs on NCh. (f) ζ-Potential of tannin–NCh mixtures (at tannin 25 wt % over NCh mass) prepared with different fractions.

We noted that the higher the *M*_w_ of the tannin fraction, the higher the *G*′ of the emulsion. This may be due to the improved hydrophilicity of NCh endowed by the introduced polyphenol, so we investigated the three-phase contact angle of the tannin–NCh complex as an indicator of its emulsification ability. The three-phase contact angle of NCh is 146.4°, which indicates an elevated hydrophobicity (Figure S7). After introduction of F1-IT, F2-IT, F3-IT, and F4-IT, the contact angle reduced to 125.37, 107.11, 96.61, and 93.71°, respectively (Figure 6c). Generally, the closer the three-phase contact angle is to 90°, the better amphiphilicity the complex is, thus having a strong ability to stabilize the oil–water interface. As previously discussed, F4-IT has a higher PD, indicating a longer chain and more hydroxyl structures that could enhance hydrophilic effect of the tannin–NCh complex made from this fraction. On the other hand, ζ-potential is typically positively correlated with the colloidal stability of nanoparticles, a relationship well elucidated by the ζ-potential results between NCh and tannins. F3-IT and F4-IT exhibit high absolute potential values, both in the form of emulsion (Figure S8) and complex (Figure 6f), which is another indication of the higher *G*′ of such samples. Moreover, the presence of tannin did not significantly change the shear thinning character of the emulsions, with viscosity gradually decreasing as the shear rate gradually increases (Figure S9). This behavior is associated with the deformation and disruption of emulsion aggregation/flocculation, as well as alignment of the high-aspect ratio stabilizing NCh.^47,48^

The stability of the emulsion was further assessed by recording its optical properties in real time over 24 h using a Turbiscan instrument. All emulsions exhibited an increase at the top and a decrease at the bottom in the delta backscattering (ΔBS) graph (Figure 6a). This behavior indicates that the oil phase separates from the water phase under gravity, with the oil moving upward and the water settling at the bottom due to their density differences. Such separation typically leads to creaming, a common phenomenon in emulsions, where the dispersed phase undergoes coalescence or flocculation and eventually phase separation. Compared with NCh alone, the addition of tannins resulted in emulsions with minimal changes in ΔBS at both the bottom and top. Moreover, the emulsion stabilized by NCh (at low contents 0.07 wt %) alone also exhibited instability in the transmittance graph (Figure S10) with an increase in transmittance at the bottom, primarily due to the ripening of gas bubbles caused by the breakage of the liquid film stabilizing the system. Although this typically occurs in foam systems, in the emulsion, the instability might be attributed to the potential breakage of the NCh film. This aligns with what we discussed above, where the addition of tannins forms a closed-packed and strong tannin–NCh complex network that greatly enhances the stability of the oil–water interface. Among these, F1-IT and F2-IT demonstrated the best emulsion stability when costabilized with NCh. The micromorphology (Figure 5c) and oil droplet size (Figure 6b) of the emulsions also showed that NCh emulsions costabilized with F1-IT and F2-IT had relatively smaller oil droplet diameters, which resulted in a slower oil droplet floating velocity and thus improved emulsion stability.

The lower *M*_w_ fractions (F1-IT and F2-IT) tend to result in smaller droplets, thereby enhancing the stability of the oil droplets in the emulsion. Previous studies have indicated that a higher concentration of emulsion stabilizer leads to increased adsorption at the oil–water interface, resulting in smaller oil droplets, and more stable emulsions.^49,50^ With a fixed NCh and tannin content for all emulsions but varied fraction types, we observed changes in the emulsion oil droplet diameter that are purely attributed to the selected tannin fraction and its corresponding properties (especially *M*_w_). Generally, ζ-potential of emulsion correlates with the oil droplet diameter, but this correlation could not be verified in our study. Some studies suggest that the interfacial composition of oil droplets changes,^51^ which indicates a need for further research to determine any potential changes in composition or organization of molecules at the interface.

To better understand the interaction between tannin–NCh and the oil–water interface, we monitored the adsorption of tannin fractions on NCh using quartz crystal microbalance with dissipation (QCM-D). First, we dip coated an NCh layer on a QCM-D sensor, and then we followed the frequency changes occurring during the tannin adsorption process (Figure 6d). After reaching the complete swelling of the chitin layer, a 1 g/L tannin solution was injected into the chamber, resulting in a significant frequency reduction that varied according to different tannin fractions. After adsorption reached a plateau at ca. 90 min, the system was rinsed with DIW to remove loosely adsorbed substances. Subsequently, the desorption of unstable adsorbed molecules caused an increase in frequency. Distinct adsorption rates were observed for the deposition of various tannin fractions on NCh with adsorption plateau taking place in the following order F1-IT < F2-IT < IT < F3-IT < F4-IT, which matches the *M*_w_ of fractions. The driving force for adsorption is the entropy gain from releasing counterions and water molecules. This is evident from QCM-D curves, where F1-IT and F2-IT exhibit fast kinetics attributed to the release of bound water. Lower *M*_w_ fractions (could also be considered as smaller macromolecules),^52^ with the highest negative charge, showed greater mobility and a stronger affinity for adsorption on positively charged NCh, when compared to their higher Mw counterparts. Higher *M*_w_ tannin fractions display lower charges and adsorb more slowly without reaching a clear plateau, suggesting possible adsorption of multilayers of tannin molecules.

Previous studies have demonstrated a continuous increase in frequency change (Δ*f*) with the increased absorption.^53−55^ Here, we calculate the adsorption mass per unit area using the Sauerbrey equation (Figure 6e), which is proportional to the decrease in the resonance frequency. The observed results (Δ*m*_IT_ < Δ*m*_F1-IT_ < Δ*m*_F3-IT_ < Δ*m*_F2-IT_ < Δ*m*_F4-IT_) indicate that all fractions adsorb irreversibly on the NCh surface, with Δ*m* being tethered to the *M*_w_ of the fractions. This confirms the favorable complexation between tannins and NCh, but it also highlights that there is a saturation point to which additional tannins do not confer further benefits on emulsion stability.

### 3.3 Oxidative Stability Enabled by the Tannin–Chitin Complex in Pickering Emulsions

**Figure 7:**
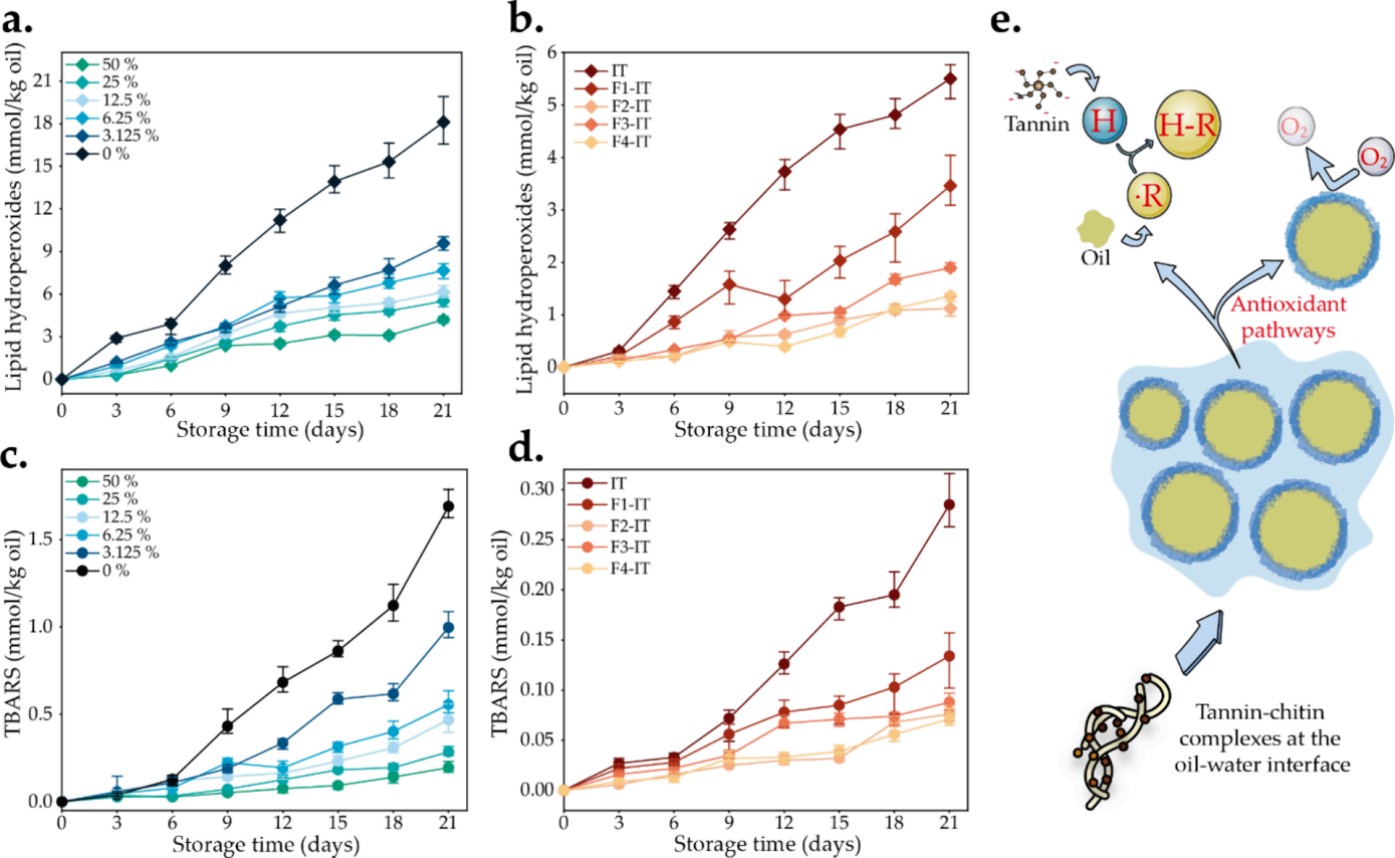
Inhibitory effect of (nonfractionated) industrial tannin content on (a) the formation of lipid hydroperoxides and (c) TBARS, and the effect of different fractions on (b) the formation of lipid hydroperoxides and (d) TBARS. (e) Schematic diagram of the antioxidant pathway of the emulsion.

The antioxidative properties of tannin in a rapeseed oil-based emulsion system were assessed by monitoring the formation of oxidation products during storage. Within the emulsion systems, the oil–water interface significantly influences the rate and extent of which lipids are oxidized, serving as the region where hydrophobic, hydrophilic, and amphiphilic molecules involved in the oxidation reaction closely interact.^56^ Consequently, the tannin to NCh ratio is anticipated to be a factor in the lipid oxidation kinetics. Over time, the levels of lipid hydroperoxides and TBARS increased in all emulsions, indicating ongoing lipid oxidation. The rate of lipid oxidation decreased with an increasing tannin content in the emulsions, which is evident in both primary (Figure 7a) and secondary (Figure 7c) reaction products. Furthermore, all tannin fractions introduced superior antioxidative effects in the emulsions compared to those of the unfractionated tannin. Notably, F2-IT demonstrated the most robust antioxidative properties (Figure 7b,d) followed by F4-IT, thus highlighting the positive effect of phenolic and flavonoid (Figure 2d,f) molecules on imparting radical scavenging. This indicates that solvent-centered fractionation can effectively retain and concentrate polyphenolic active components in the fractions, thereby enhancing their biological activity and antioxidant capacity. Nevertheless, oxidation can be drastically diminished by the formation of a physical, interlocked layer at the oil–water interface,^57^ which we introduced by exploiting the complexation between negatively charged tannin fractions and positively charged chitin colloids. Fractionation leads to controlled tannin adsorption onto chitin, resulting in varying barrier properties, depending on the *M*_w_ and PD of each tannin fraction, thus also effectively slowing down the oxidation products by blocking the contact of oil with oxidants such as O_2_, hydrogen peroxide, and transition metals in the aqueous phase.

Based on the above results, a schematic model was proposed to explain the mechanism of how tannins contribute to enhancing the antioxidative properties of the Pickering emulsion (Figure 7e). Tannins can effectively bind to active oxygen radicals (R·) by donating a hydrogen atom, thereby converting R· to the less reactive H-R. This is due to the abundant presence of hydroxyl groups within the tannin chemical structure, which acts as a proton exchanger.^58^ Moreover, the ability of tannin fractions/NCh to better stabilize the oil–water interface when compared to pure NCh, attributed to an adjustment in the contact angle of the complex, creates physical barriers that decrease the gas permeation to the oil phase via the stabilization layer. The content of polyphenolic active ingredients is positively correlated with the antioxidant properties of the emulsions (Figure S11). Interestingly, the ability of tannin to effectively adsorb on chitin, which indirectly indicates strong binding, has a positive correlation with the antioxidant capacity. Overall, our fractionation process enabled fractions of controlled properties, but more than that, it enabled us to understand what factors affect the most the properties, of emulsions made from polyphenols. We indicate that molar mass and associated interaction capacity are driving parameters to manipulate emulsion microstructure and properties. This is applicable in many industries, for example, food and cosmetics.

## 4 Conclusions

ITs were effectively fractionated, via a solvent-centered fractionation process, into fractions with high homogeneity and adjustable *M*_w_. A sequence of ACN-ACE-MeOH–EtOH was used and led to tannin fractions with DIs below 2, which remarkably improved their applications in functional Pickering emulsions costabilized with low content (0.07 wt %) of chitin nanofibrils (NCh). Tannin fractions rapidly bind NCh at the oil–water interface, thus improving the emulsions' microstructure and stability, while introducing antioxidant properties (TBARS and lipid hydroperoxides formation reduced by five times). *M*_w_ (ca. 1500–2500 g/mol) of the fractions has a direct relationship with the emulsion droplet size (*d*_(0,5)_ = 2.4–3.7 μm). Our systematic fractionation process, followed by a detailed chemical characterization of each fraction, opens several possibilities for practical use. For example, via this process, we prepared fractions with low and high condensed tannin contents, as well as fractions that are enriched by flavonoids. Our residue material displays low polyphenol content, and may not be suitable for emulsion, but it could find use in applications where minerals and carbohydrates are needed. Overall, this study demonstrates the effectiveness of solubility-driven fraction in reducing chemical heterogeneity of ITs, and it explores practical applications of fractionated tannin to highlight the benefits of having well-defined polyphenol building blocks in high relevant end uses, such as emulsions. We do recognize that strategies for solvent recovery, recycling, and overall optimization are required before this method can be considered for large-scale implementation. Ideally, fractionation would be integrated with already existing tannin extraction facilities, by leveraging existing infrastructure to increase the value-added of their product portfolio.

## Supporting Information Available

Preparation method of chitin nanofibrils (NCh); *M*_w_, *M*_n_, and Dis of soluble and insoluble parts of tannin in different solvents; DI comparison with other fractionation methods; element content; GPC curves; picture of fractions; FTIR patterns; ^31^P NMR spectrum; AFM image; picture of emulsions; three-phase contact angle; zeta-potential of emulsions; viscosity of emulsions; and Turbiscan transmittance curves (PDF)
