# Supplementary material for: Refined Industrial Tannins via Sequential Fractionation: Exploiting Well-Defined Molecular Structures for Controlled Performance in Pickering Emulsions Costabilized with Chitin Nanofibrils
Source: ACS Sustain Chem Eng. 2024 Nov 20;12(49):17878–90. doi: 10.1021/acssuschemeng.4c07769 (PMC11633650; doi:10.1021/acssuschemeng.4c07769)
Supplement: Supplementary file 1 — sc4c07769_si_001.pdf [file sc4c07769_si_001.pdf]

Supporting information:

Refined industrial tannins via sequential fractionation: exploiting well defined molecular structures for controlled performance in Pickering emulsions co-stabilized with chitin nanofibrils

Weitong Wang[†], *Ya Zhu* [†], *Monika Österberg*[†]* *and Bruno D. Mattos* [†]*

[†]*Department of Bioproducts and Biosystems, School of Chemical Engineering, Aalto University, P.O. Box 16300, FIN-00076 Aalto, Espoo, Finland.*

*Monika Österberg. E-mail: monika.osterberg@aalto.fi. Tel: +358505497218.

*Bruno D. Mattos. E-mail: bruno.mattos@aalto.fi. Tel: +358503582138.

Number of Pages: 16

Number of Figures: 11

Number of Tables: 3

1. Methodologies, encompassing purification and deacetylation of NCh

NCh was obtained from fresh crabs (Callinectes sapidus) that were acquired in the local market (Helsinki harbor, Finland). Steps yielded partially deacetylated chitin (DE-chitin, degree of deacetylation to be ~27%) are as below:

The obtained DE-chitin was redispersed into DIW (pH 3 with acetic acid) at 0.2 wt % solid content using a high-speed blender (T-25 Ultra-Turrax Digital Homogenizer, IKA, Germany) operated at room temperature for 5 min, which fully protonated the obtained amine groups. Afterward, microfluidization (M-110P, Microfluidics Inc., Newton, MA) was used to disintegrate the DE-chitin into NCh with a single pass at a pressure of 1200 bar. The obtained NCh was centrifuged at 11000 rpm for 5 min to remove large particles, and the supernatant was collected, concentrated (0.7 wt %, pH 3), and stored at 8 °C for further use. The average aspect ratio of NCh was calculated to be ~20 by counting the length and width from at least 30 nanofibers from Figure S5.

The protonation degree of NCh at the emulsion pH was calculated by *Henderson-Hasselbalch* equation:

$$pH = pKa + \log((A^-)/(HA))$$

Where (A^-) is the concentration of the deprotonated form (unprotonated), (HA) is the concentration of the protonated form. The pKa values of NCh is 6.3 with a 27% degree of deacetylation, the emulsion pH is around 3.3.

The equation to calculate the ratio of protonated:

$$(A^-)/(HA) = 10^{(pH - pKa)}$$

Thus, the protonation degree of NCh = (HA)/ [(HA)+ (A-)] = 99.9%.

2. ^{31}P NMR Analysis

2.1. Preparation of NMR Solutions

The internal standard (IS) solution was prepared using e-NHI at a concentration of 9.27 mg/mL in $CDCl_3$. Relaxation agent (Cr(III) acetylacetonate) was dissolved in $CDCl_3$ at a concentration of 5.6 mg/mL. The NMR solvent mixture was stored over molecular sieves under an argon atmosphere.

2.2. Phosphitylation Procedure

Firstly, weigh 30 mg of tannin into a 2 mL glass vial containing a mini magnetic stirrer and dried overnight at a vacuum oven. Then add the following solutions in sequence according to the specified amounts:

1. 0,150 ml DMF (N,N-Dimethylformamide, SigmaAldrich),

2. 0,100 ml Pyridine (SigmaAldrich),

3. 0,200 ml IS (e-NHI, *N*-Hydroxy-5-norbornene-2,3-dicarboxylic acid imide, Merck),

4. 0,050 ml Cr(III) acetylacetonate (Chromium(III) 2,4-pentanedionate, SigmaAldrich),

5. 0,300 ml $CDCl_3$ (Chloroform D, SigmaAldrich),

6. 0,150 ml phosphitylating reagent (2-chloro-4,4,5,5-tetramethyl-1,3,2-dioxaphospholane, SigmaAldrich),

Then, dissolve sample fully by 10 min magnetic stirring, and transfer into an NMR tube.

2.3. ^{31}P NMR Spectroscopy

The ^{31}P NMR spectra were recorded on a Bruker NMR Spectrometer AV III 400 spectrometer. The probe temperature was set at 25 °C. Typical spectral parameters for quantitative studies were followed with 90° pulse angle. All chemical shifts reported are relative to the reaction product of water with Cl-TMDP, which has been observed to give a sharp signal in pyridine/$CDCl_3$ at 132.2 ppm.

Table S1. Weight average molecular weight (Mw), number average molecular weight (Mn) and Dispersity index (DI) of both soluble and insoluble portions of IT.

		M_n	M_w	PDI
ACN	I	726.7	2370.8	3.26
	S	977.9	1445.5	1.47
ACE	I	625.1	1770	2.83
	S	581.3	1360.2	2.34
MeOH	I	670.9	2270.1	3.38
	S	598.3	1569.7	2.62
EtOH	I	768.8	3889.1	5.06
	S	585.8	1550.8	2.65

ACN: acetonitrile, ACE: acetone, MeOH: methanol, EtOH: ethanol.

I: insoluble, S: soluble.

Table S2. Comparisons of PDIs for the fractions in this study with other studies.

Raw material	Fractionation method	PDI	Reference
Tannin extraction of black wattle (Acacia mearnsii) bark	Sequential fractionation (Soxhlet extraction)	**1.47~1.99**	**This work**
Tannin extraction of black wattle (Acacia mearnsii) bark	Sequential fractionation (Soxhlet extraction)	2.41~4.55	Missio, et al., 2017
Tannin extracted from shoots and leaves of *L. leucocephala*	Chromatography method (Sephadex G-25 gel)	2~5	Saminathan, et al., 2015
Tannin extraction of black wattle (Acacia mearnsii) bark	Solid-liquid extraction	2.99~3.84	Sepperer, et al., 2019

Table S3. Elemental compositions of various biochar.

	C (%)[a]	N (%)[a]	H (%)[a]	S (%)[a]	O (%)	Mg (%)[b]	K (%)[b]	Ca (%)[b]
IT	50.98	0.48	5.26	0.00	42.55	0.11	0.53	0.09
F1-IT	56.51	1.10	5.19	0.00	35.77	0.37	0.75	0.31
F2-IT	57.51	0.10	5.12	0.00	36.74	0.03	0.50	0.00
F3-IT	51.77	0.70	5.16	0.00	41.28	0.06	1.02	0.01
F4-IT	51.78	0.41	5.05	0.00	42.65	0.00	0.10	0.01
Residue	44.47	1.17	4.91	0.00	49.28	0.01	0.16	0.00

a: Determined by elemental analysis (weight%), O% = 100% - (C% + N% + S% + H% + Si% + Al% + Ca%).

b: Determined by SEM-EDX (weight%).

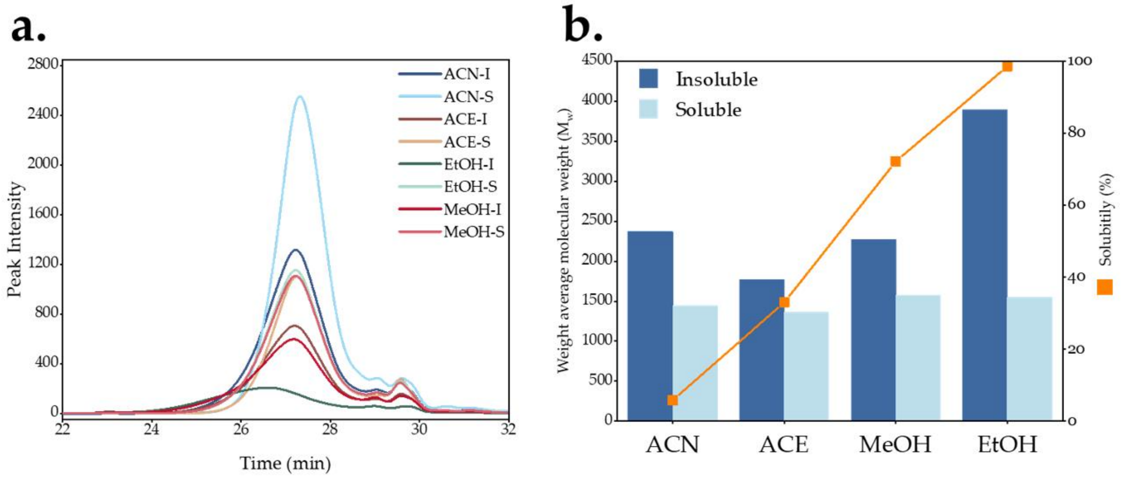

Figure S1. The GPC curves (a.) and Weight average molecular weight (Mw) of tannin's insoluble and soluble part in different solvents (Acetonitrile, acetone, methanol, ethanol) both with the solubility (b.).

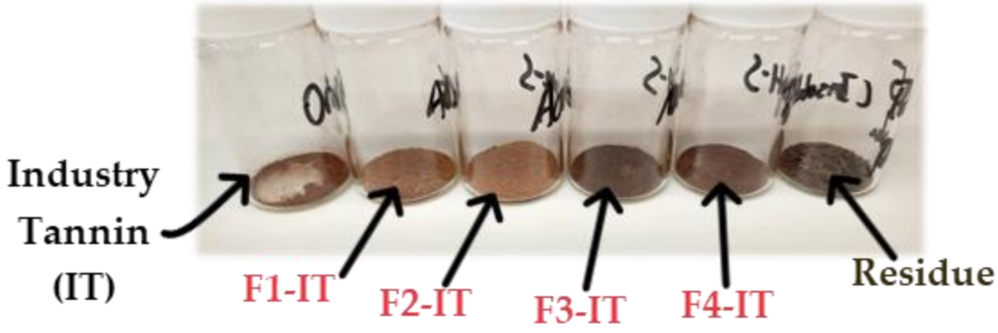

Figure S2. Picture of IT, Fx-IT and residue; fractions show different colors, which may related to the different chemical compositions.

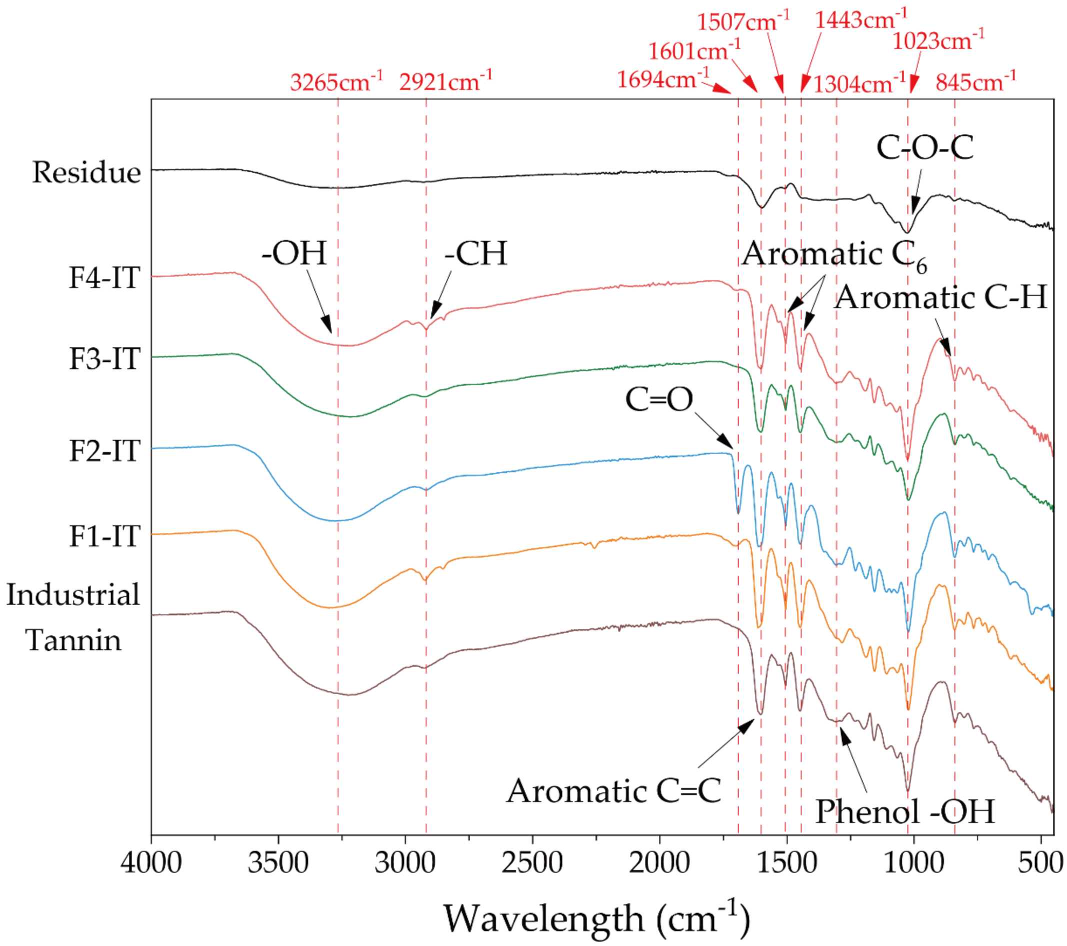

Figure S3. The FTIR curves of IT, Fx-ITs, and residue.

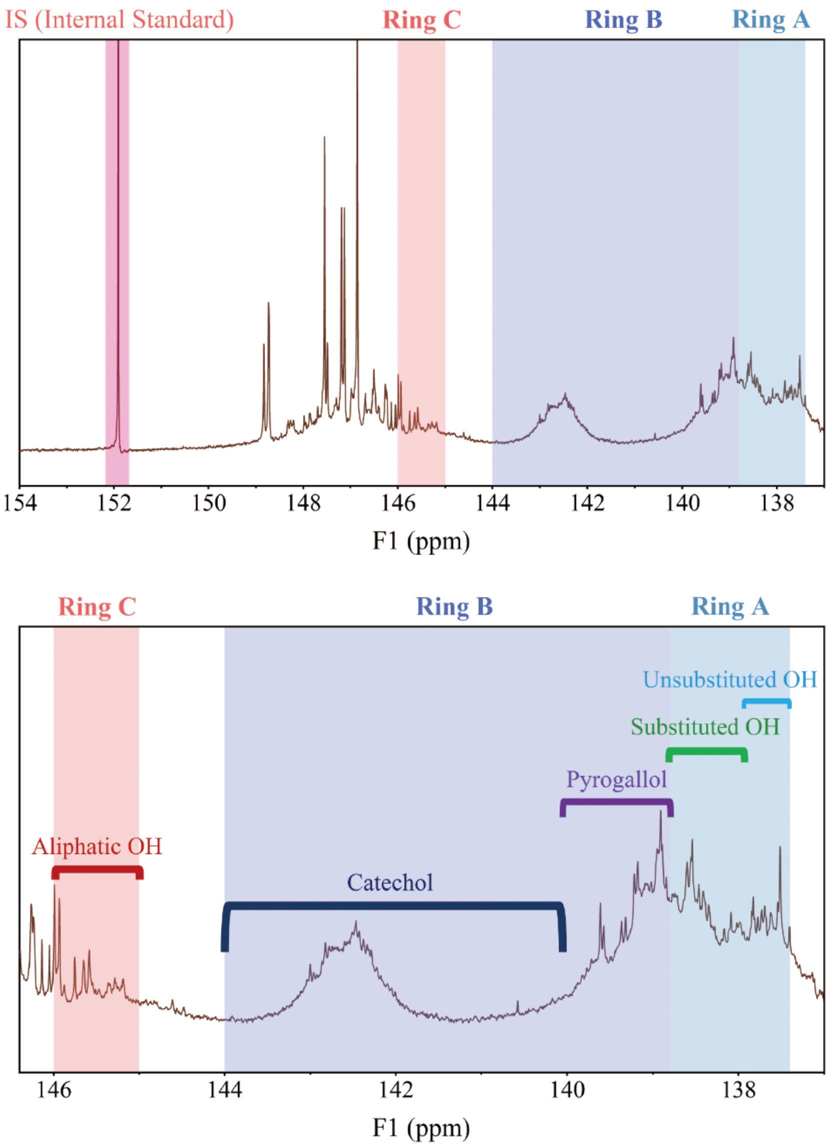

Figure S4. ^{31}P NMR spectrum of Industrial Tannin from *Accia mearnsii*.

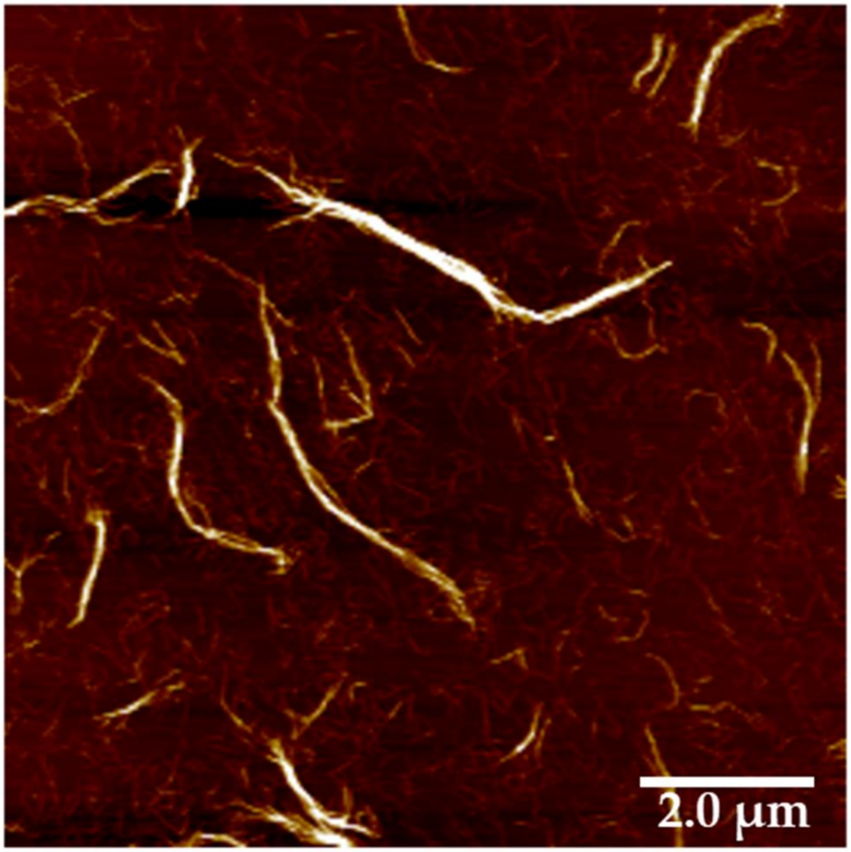

Figurer S5. Atomic force microscopy (AFM) images of chitin nanofibrils (NCh), the aspect ratio of NCh is approximately 20.

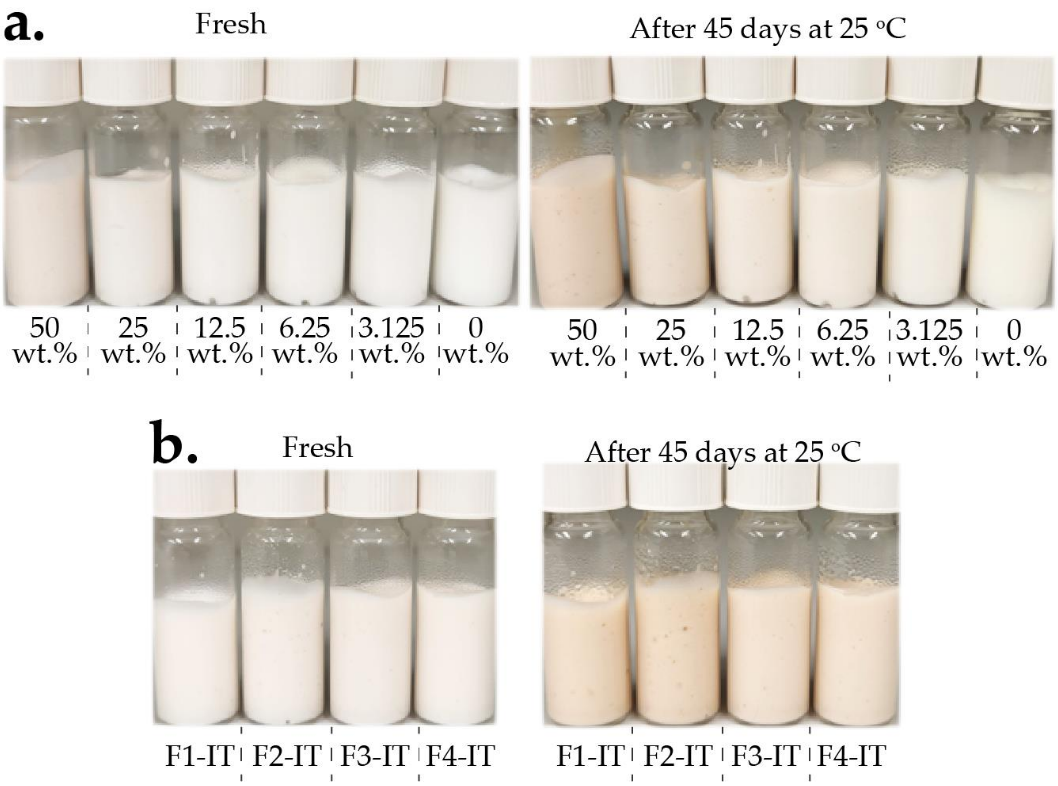

Figurer S6. Stability of fresh and aged Pickering emulsions prepared with **a)** varying contents of (non-fractionated) industrial tannins and **b)** fractionated tannins at 25 wt%.

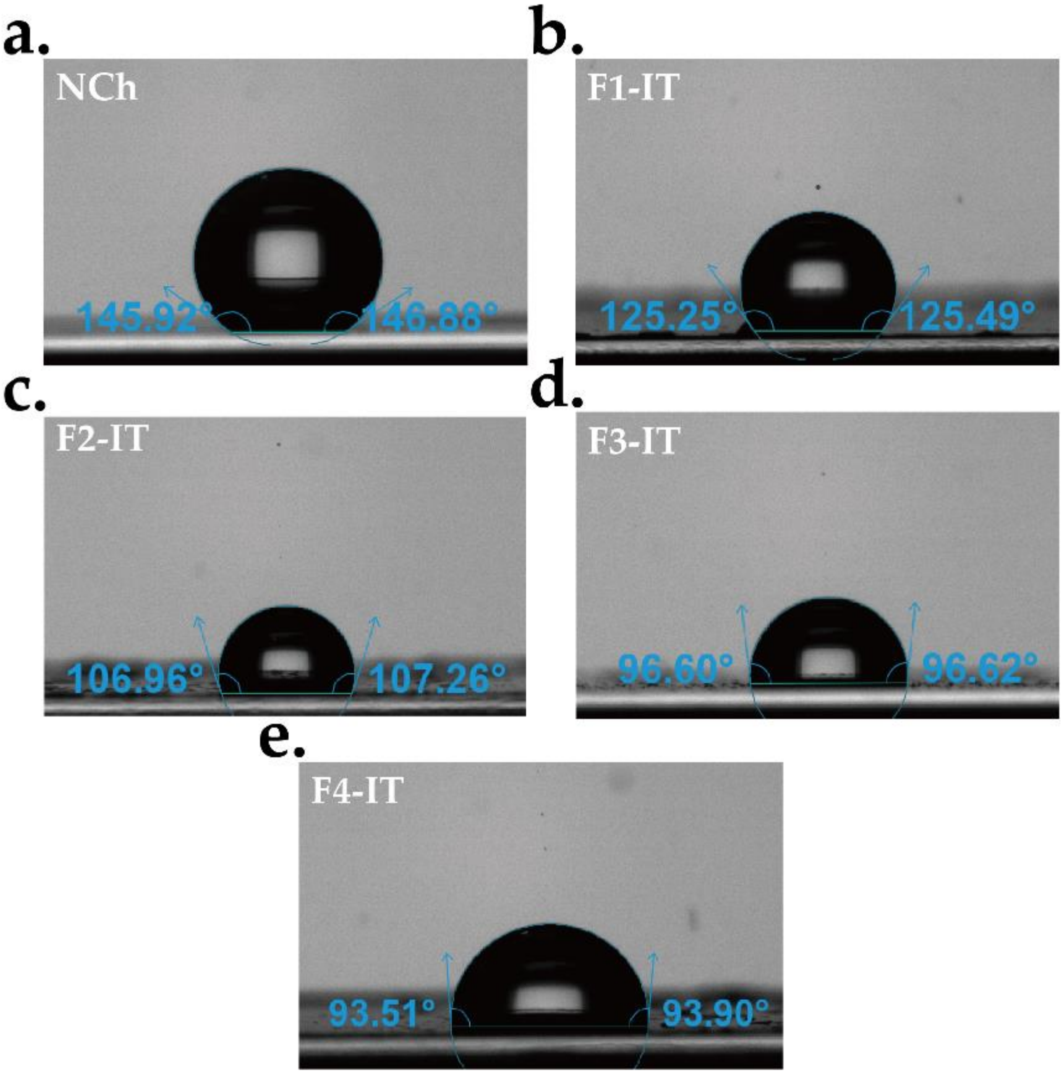

Figure S7. Three-phase contact angle of **a)** pure NCh, and NCh complexed with **b)** F1-IT, **c)** F2-IT, **d)** F3-IT, **e)** F4-IT.

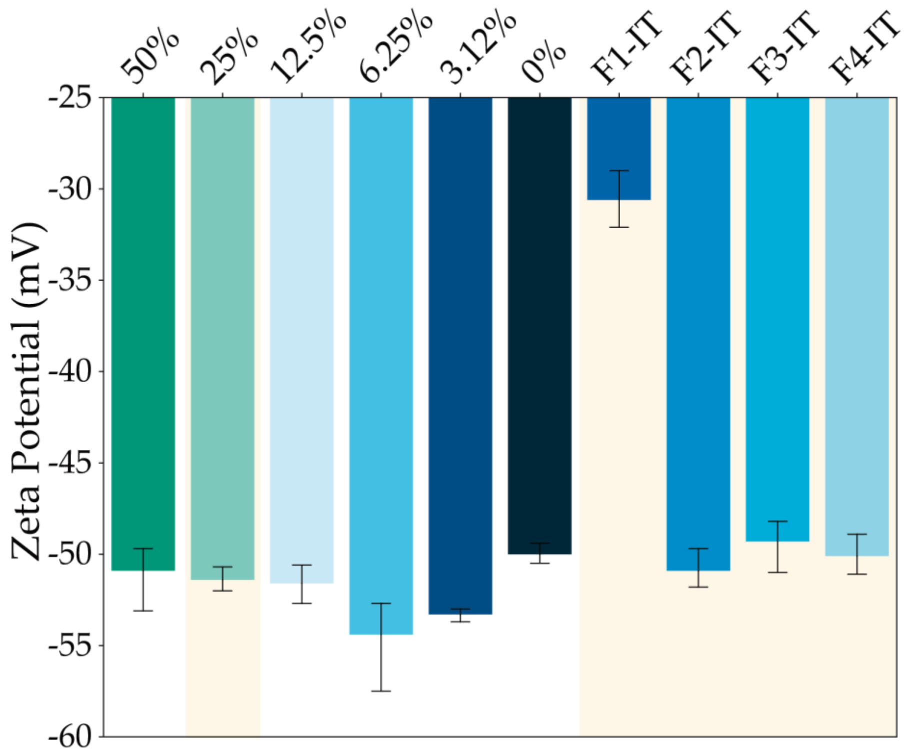

Figure S8. ζ-potential of various Pickering emulsions stabilized with different content of IT or different type of fractions.

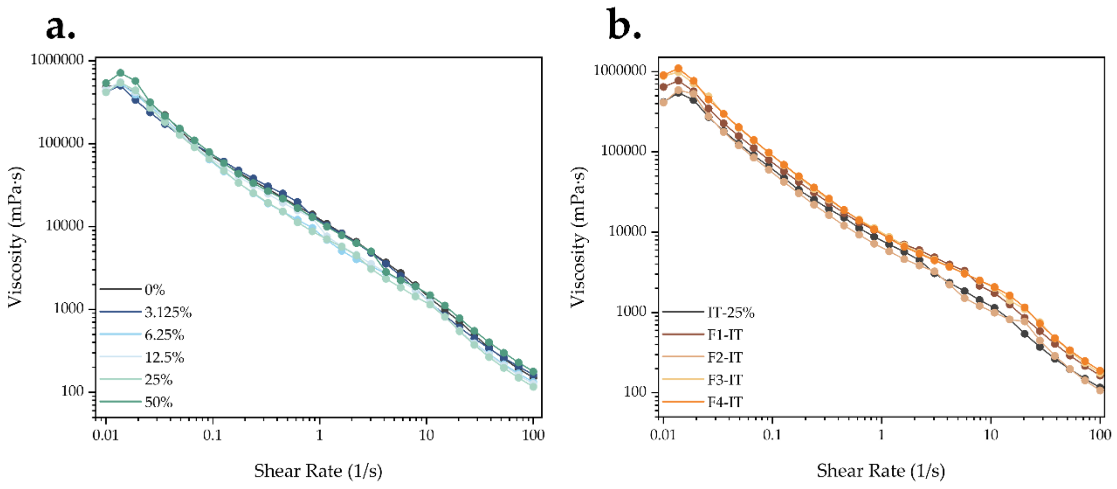

Figure S9. The viscosity of (b.) emulsion with various mass ratios of IT to NCh and (d.) emulsion stabilized by different fractions.

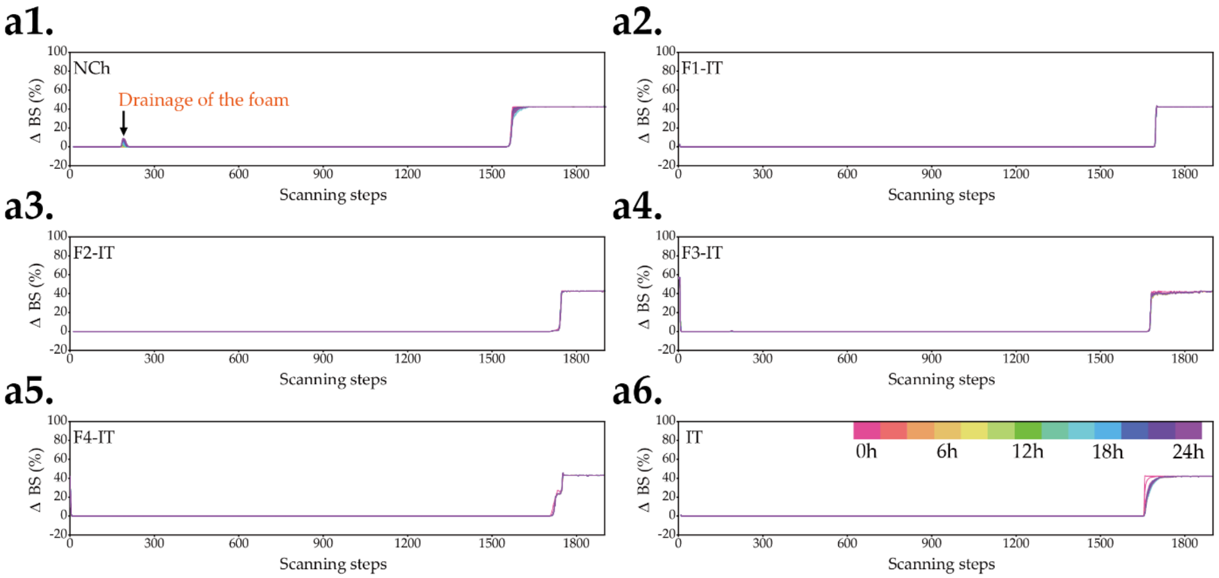

Figurer S10. Turbiscan transmittance curves of emulsions stabilized without tannin (pure NCh) **a1)**, and with **a2)** F1-IT, **a3)** F2-IT, **a4)** F3-IT, **a5)** F4-IT and **a6)** IT.

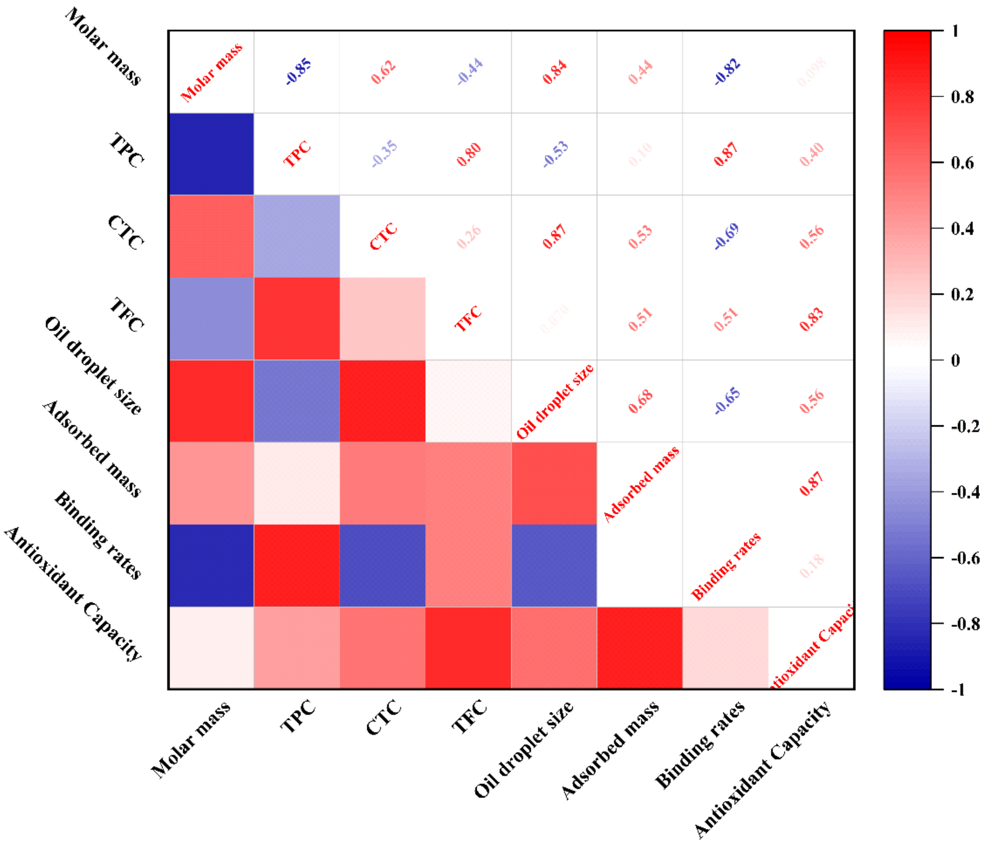

Figure S11. Correlation plots showing Pearson correlation values of the molecular weight of tannin and its fractions, emulsion droplet size, binding rate with NCh, and adsorption amount on NCh surface (Obtained from the QCM-D data), as well as the correlations values of polyphenolic active ingredients and antioxidant properties.

REFERENCES

1. A. L. Missio, B. Tischer, P. S. B. dos Santos, C. Codevilla, C. R. de Menezes, J. S. Barin, C. R. Haselein, J. Labidi, D. A. Gatto, A. Petutschnigg and G. Tondi, *Separation and Purification Technology*, 2017, **186**, 218-225.
2. M. Saminathan, C. C. Sieo, N. Abdullah, C. M. Wong and Y. W. Ho, *J Sci Food Agric*, 2015, **95**, 2742-2749.
3. T. Sepperer, F. Hernandez-Ramos, J. Labidi, G. J. Oostingh, B. Bogner, A. Petutschnigg and G. Tondi, *Industrial Crops and Products*, 2019, **139**.
